# Does Native Vitamin D Supplementation Have Pleiotropic Effects in Patients with End-Stage Kidney Disease? A Systematic Review of Randomized Trials

**DOI:** 10.3390/nu15133072

**Published:** 2023-07-07

**Authors:** Nathan G. Pilkey, Olivia Novosel, Angélique Roy, Tristin E. Wilson, Jaya Sharma, Sono Khan, Sanjana Kapuria, Michael A. Adams, Rachel M. Holden

**Affiliations:** 1 Department of Biomedical and Molecular Sciences, Queen’s University, Kingston, ON K7L 3N6, Canada; nathan.pilkey@mail.mcgill.ca (N.G.P.); 18omn@queensu.ca (O.N.); 21tew@queensu.ca (T.E.W.); 19jks5@queensu.ca (J.S.); sono.khan@queensu.ca (S.K.); 20sk6@queensu.ca (S.K.); adams@queensu.ca (M.A.A.); 2 Bracken Health Sciences Library, Queen’s University, Kingston, ON K7L 3N6, Canada; angelique.roy@queensu.ca; 3 Department of Medicine, Queen’s University, Kingston, ON K7L 3N6, Canada

**Keywords:** vitamin D, end-stage kidney disease, hemodialysis, parathyroid hormone, phosphate, calcium

## Abstract

Vitamin D has been shown to have multiple pleiotropic effects beyond bone and mineral metabolism, with purported roles in cardiovascular disease, cancer, and host immunity. Vitamin D deficiency is common in patients with end-stage kidney disease (ESKD); however, current clinical practice has favored the use of the active hormone. Whether vitamin D deficiency should be corrected in patients with ESKD remains unclear, as few randomized trials have been conducted. In this systematic review, we summarize the current evidence examining whether vitamin D supplementation improves outcomes, beyond mineral metabolism, in patients with ESKD. Data from randomized controlled trials of adults with ESKD were obtained by searching Ovid MEDLINE, Embase, the Cochrane Central Register of Controlled Trials, and the Web of Science Core Collection from inception to February 2023. Twenty-three trials composed of 2489 participants were identified for inclusion. Data were synthesized by two independent reviewers and summarized in tables organized by outcome. Outcomes included measures of mortality, cardiovascular disease, inflammation, muscle strength/function, nutrition, patient well-being, and outcomes specific to ESKD including erythropoietin usage, pruritus, and dialysis access maturation. The Cochrane risk of Bias Tool (RoB 2, 2019) was used to assess study quality. Overall, our findings indicate a minimal and varied benefit of native vitamin D supplementation. From the largest studies included, we determine that vitamin D has no demonstrated effect on patient-reported measures of well-being or utilization of erythropoietin, nor does it change levels of the inflammation biomarker *C*-reactive protein. Included trials were heterogeneous with regards to outcomes, and the majority studied small participant populations with a relatively short follow-up. We conclude that vitamin D supplementation corrects vitamin D deficiency and is safe and well-tolerated in humans with ESKD. However, it is not clear from clinical trials conducted to date that a causal pathway exists between 25(OH)D and pleiotropic effects that is responsive to vitamin D treatment.

## 1. Introduction

Vitamin D may have actions beyond the expected effects on mineral homeostasis and bone disease. Local production of 1.25(OH)_2_D in cells of the immune system, gastrointestinal system, breast, and skin has expanded the understanding of the vitamin D endocrine system to include potential pleiotropic effects that might benefit cardiovascular (CV) disease, host immunity, glucose homeostasis, cancer, and inflammation [1,2].

Vitamin D insufficiency is a recognized component of the chronic kidney disease mineral bone disorder (CKD-MBD). Vitamin D insufficiency, defined by low levels of 25(OH)D, is very common in patients with end-stage kidney disease (ESKD) and is due, in part, to comorbidities, factors related to sun exposure, restricted diets, and urinary losses [3,4]. Despite the high prevalence of vitamin D insufficiency in this population, there is no clear guidance with regards to surveillance and treatment.

Vitamin D status is assessed by total 25(OH)D levels. In a systematic review of cohort studies, a single 25(OH)D level in the sufficient range was associated with lower all-cause and CV mortality in patients with ESKD [5]. Whether a low 25(OH)D is a predictive factor that is modifiable and a target to treat, or primarily a biomarker that identifies high risk, or possibly both, is not known [6]. The vast majority of evidence related to vitamin D supplementation in ESKD is limited to observational studies where a causal relationship with clinical outcomes cannot be determined. Only randomized trials can obviate the significant confounding associated with studies of vitamin D supplementation in patients with ESKD.

The utility of treating vitamin D insufficiency in patients with ESKD is an area of controversy. We see that 25(OH)D_3_ undergoes hydroxylation to the active hormone, 1.25(OH)_2_D_3_, by CYP27B1 in proximal convoluted tubule cells of the kidney. This process, regulated by parathyroid hormone (PTH) and fibroblast growth factor-23, is likely responsible for calcium and phosphate homeostasis [7]. To date, current clinical practice has favored the use of the active hormone as it was thought to provide the vitamin D needs of the patient with ESKD, and supplementation with native vitamin D was not strongly supported by guidelines [8,9]. Whether to replace vitamin D in patients with minimal kidney function is uncertain as few clinical trials have been conducted in this patient group to inform practice. The identification of CYP27B1 in extra-renal tissues has renewed interest in supplementing patients with ESKD with native vitamin D for its potential benefits beyond mineral homeostasis [10].

In this systematic review of randomized trials, we sought to evaluate the potential pleiotropic effects of vitamin D supplementation on outcomes beyond those related to CKD-MBD laboratory parameters in patients with ESKD.

## 2. Methods

This systematic review was conducted according to the guidelines for Preferred Reporting Items for Systematic Reviews and Meta-analyses [11]. The review protocol was not registered at the time of study initiation. We used PICOS (population, intervention, comparison, outcomes, and settings) (Table 1) to guide our review question as follows: in randomized trials of patients with ESKD requiring dialysis, does vitamin D replacement modify outcomes beyond those related to mineral metabolism?

### 2.1. Eligibility Criteria

We applied the following inclusion criteria: parallel, randomized trials of adult patients with ESKD receiving dialysis of any ethnicity that involved vitamin D supplementation with a control group (placebo or no-treatment control group). We did not place limits on the duration of the study, nor did we specify outcomes. Trials published up until February, 2023 were considered.

### 2.2. Intervention Types

All studies in which participants received a native vitamin D supplement—either cholecalciferol, ergocalciferol, or calcifidiol—compared with a non-supplemented group were considered for inclusion. Co-interventions were permissible.

### 2.3. Types of Outcome Measures Reported

To evaluate all potential pleiotropic effects of vitamin D, reported data from any trial were considered. We excluded studies where the only reported measures related to laboratory parameters of CKD-MBD including 25(OH)D levels. However, for each of the included trials we report the 25(OH)D levels at baseline and study end. We included studies where clinically relevant laboratory measures were the primary outcome if the measure was not directly related to mineral homeostasis. The primary outcome was the change in the outcome measure (e.g., imaging test, clinical event, and laboratory measure) from baseline to study end.

### 2.4. Search Strategy

A comprehensive search strategy was developed by a librarian to identify relevant published trials as well as unpublished work (gray literature) in pre-prints, conference materials, abstracts, and clinical trial registries. An initial search of PubMed and Google Scholar was conducted to identify relevant search terms and subject headings for inclusion. Embase (Ovid) was used to develop the search strategy, which was reviewed by a second librarian at Queens University Library. The final search was conducted in MEDLINE (Ovid), EBM Reviews for Cochrane Central Register of Controlled Trials (CENTRAL via Ovid), Web of Science Core collections, ProQuest Dissertation and Theses Global, and medRxiv. All adapted database search strategies were peer-reviewed.

Given the high volume of records resulting from the combination of the vitamin D and kidney disease concepts, the Cochrane Highly Sensitive Search Strategy was employed to identify and screen relevant trials in Embase [12]. The MEDLINE search was conducted using the same Cochrane Highly Sensitive Search Strategy for identifying randomized controlled trials, adapted for the MEDLINE database [13]. A combination of the two database searches was employed for searching the Web of Science collection. Databases were searched from inception to 2 May 2022 with an update performed across all databases to retrieve records from this period up to 31 January 2023.

After the 31 January 2023 update, the total number of search results from all databases and information sources searched was 9632. The number of records after removing duplicates in Covidence systematic review software was 6519. The total number of records identified for each database and information source is provided in Appendix A. Search strategies for each database and information source searched are presented in Appendix B.

### 2.5. Study Selection

Five authors (NP, ON, TW, JS, and SK) independently screened the studies, and each abstract was reviewed independently by two reviewers. Clearly irrelevant studies were excluded. Full texts of remaining studies were obtained, screened, and excluded if irrelevant (NP and ON). Where disagreements occurred, they were resolved by a third review (RH). All studies were screened using Covidence systematic review software.

### 2.6. Data Items

Data were extracted by two independent reviewers (NP and ON): first author’s name, year of publication, country, and number of centers involved. The study design was assessed and included population and specific inclusion criteria, method of assigning patients to different treatments, specific details of the intervention and control arms, length of treatment, length of follow-up, number of randomized versus analyzed, and number lost to follow-up. Summary data regarding the age of participants and the outcome measures were obtained. No formal assessment of agreement between raters was obtained.

### 2.7. Data Synthesis and Analysis

The data were synthesized in a narrative form and then sub-grouped based on themes of outcomes. These themes included mortality, CV disease (surrogate markers (imaging and laboratory) and clinical events), inflammation (*C*-reactive protein (CRP)), metabolic outcomes (lipids and albumin), musculoskeletal outcomes (grip strength, bone mineral density (BMD), and fracture), patient well-being outcomes (health-related quality of life (HRQOL) and depression), and outcomes specific to dialysis/ESKD (e.g., anemia management, pruritis, and arteriovenous fistula maturation). Study descriptions and clinical characteristics are summarized by summary tables and text. Overall, we sought to summarize the direction of any observed effects of vitamin D treatment across studies. Based on substantial heterogeneity between studies, meta-analysis was not performed.

### 2.8. Assessment of Risk of Bias

The risk of bias for each study was assessed using the Cochrane Risk of Bias tool version 2 [14] by two review authors (NP and ON). A third reviewer (RH) resolved disagreements. We used the following six criteria to determine whether the risk was low, high, or had some concerns: randomization process, effect of assignment to intervention, effect of adherence to intervention, missing outcome data, measurement of outcome, and selection of reported results.

### 2.9. Measurement of Treatment Effect

Many trials reported continuous measures at baseline and study end and determined the treatment effect using tests of statistical significance. Some trials reported event rates at study end (e.g., mortality and CV events). For ease of reporting, we categorized trials into tables that encompassed the themes described above. Some (n = 11) trials are reported in more than one table if the reported outcomes in the study encompassed more than one theme (e.g., Wang et al. reported CRP and psychological health) [15]. Measures of statistical significance of treatment effect were reported directly from the respective studies.

### 2.10. Safety

The trials were examined for reported events of hypercalcemia and vitamin D toxicity.

## 3. Results

### 3.1. Study Selection

**Figure 1:**
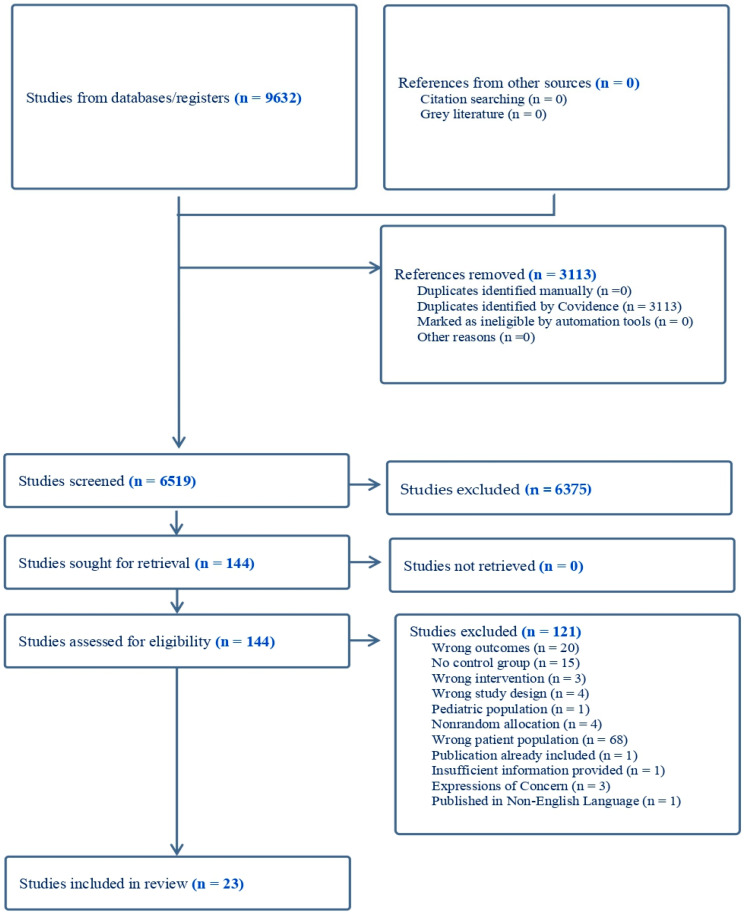
PRISMA flow chart of study review and selection.

The results of the search are presented in Figure 1. Of the 144 studies included for full-text review, 23 studies were conducted in patients with ESKD and included in this review.

### 3.2. Study Characteristics

All 23 studies selected for inclusion in this review were randomized trials published in English. One trial was excluded as the body of text and abstract were not in English [16]. Three trials were excluded due to multiple expressions of concern published by journals [17,18]. One study was excluded as data were presented by sub-categories within treated and control groups, based on baseline 25(OH)D level, and the number of patients per stratification was not reported [19]. Two studies that were included were only available in abstract form [20,21].

The 23 studies included in this review are presented in Table 2. The studies are presented in alphabetical order by the surname of the first author. Overall, 2489 participants were included in these studies (1281 randomized to vitamin D and 120mt8 randomized to control). Nineteen trials were randomized, and placebo-controlled, whereas the control arm was standard of care in four trials [20,21,22,23]. The geographical regions where the trials were conducted included Europe (n = 7), North America (n = 6), Asia (n = 2), Gulf States (n = 4), South America (n = 2), and Australia (n = 2). The majority of trials were conducted in a single center, whereas six trials included more than one center, and in four trials it was not specifically reported. Overall, the sample size studied was fewer than 100 participants in 19 trials and was 60 or fewer in 12 of these. In 14 trials, participants were randomized only if vitamin D insufficiency was present, whereas baseline vitamin D status was not considered for eligibility in 9 trials. Elevated PTH and pruritis were required for inclusion in two studies [24,25]. The vitamin D intervention included cholecalciferol (n = 20), ergocalciferol (n = 2), and calcifediol (n = 1). In trials of cholecalciferol, the most common dosing regimen was 50,000 units weekly, with some studies employing a step-down to a lower weekly dose once a sufficient 25(OH)D level was obtained. One three-week study provided 200,000 IU weekly [24], and the study of Khajehdehi reportedly provided 50,000 units daily for 3 months [26]. The two studies conducted in the United States using ergocalciferol employed similar dosing regimens (50,000 units weekly with a step-down to monthly) [27,28]. The study of calcifediol provided 40 mc thrice weekly [22]. Follow-up ranged from 3 weeks to 24 months. The primary outcome of the trial is presented in Table 1, and the timing of the various outcome measures is presented at baseline and study end. In two trials, the duration of follow-up for ascertainment of clinical outcomes extended beyond the treatment duration [28,29]. We report only on measured outcomes and/or clinical events not directly related to laboratory measures of CKD-MBD. A component of laboratory assessment of CKD-MBD or 25(OH)D levels was the primary outcome of the trial in seven cases [21,23,24,25,27,30,31].

### 3.3. Risk of Bias Assessment

In Table 3, we present the quality measures of the studies. There was a low risk of bias across all parameters in the studies by Bhan, Brimble, Hewitt, Miskulin, Seibert, and Singer. Otherwise, bias due to the effect of assignment to intervention and adhering to intervention was deemed to be high if not adequately reported. A number of trials were deemed to have concerns or be at high risk of bias based on missing outcomes if drop-out from the study was unacceptably high or, as in several cases, not specifically reported upon. Several studies were deemed to be at high risk of bias across several of the assessed domains.

### 3.4. Vitamin D Supplementation and Mortality Outcomes

In the four trials that reported mortality outcomes, the treatment duration ranged from 4 to 24 months; however, follow-up in two of the trials extended beyond the treatment period (Table 4) [22,27,29,40]. The formulation of vitamin D included ergocalciferol (n = 1), the pre-hormone calcifidiol (n = 1), and cholecalciferol (n = 2). Mortality was only pre-specified within the composite primary outcome in the study where supplementation was provided by the pre-hormone, calcifediol, in an open-label fashion [22]. Although this phase-III multicenter study was the largest trial, and included 284 patients receiving HD, the study did not reach the projected sample size to address the primary outcome due to funding issues. Over 24 months, supplementation with calcifediol did not prevent mortality compared with standard care [22]. However, a large proportion of study patients did not achieve vitamin D sufficiency (36% in the treatment group versus 11% in the placebo group). Bhan et al. conducted a three-arm, 12-week trial in incident patients receiving HD that compared weekly ergocalciferol to monthly administration and placebo [27]. A large separation between the groups in terms of 25(OH)D levels was achieved. The mortality outcome was assessed at 12 months where a trend towards a benefit of monthly ergocalciferol supplementation on mortality (*p* = 0.08) was observed as well as a non-significant trend favoring the combination of weekly and monthly ergocalciferol arms compared to placebo (HR 0.28; 95% CI, 0.07 to 1.19, *p* = 0.07) [27]. Brimble et al. found a significant effect of cholecalciferol treatment on all-cause mortality (12% in the vitamin D group versus 39% in the placebo group (*p* = 0.004)) and death from CV cause (3% in the vitamin D group versus 19% in the placebo group, *p* = 0.03) in patients receiving PD [29]. At the end of this trial, 96% of participants were replete (defined as 25(OH)D level ≥ 50 nmol/L) compared to only 14% of the placebo group indicating successful correction of vitamin D deficiency [29]. However, the number of events was small and the length of follow-up for the mortality outcome extended beyond the 1-year treatment with vitamin D. The trial by Hewitt et al. included 68 vitamin D insufficient patients with ESKD on either HD or PD and was designed to determine the impact of 12 months of cholecalciferol supplementation on hand grip strength and quality of life. Only one participant died during the follow-up and, overall, adverse events were similar between groups [33]. No difference between the groups in rates of hospitalization was reported in any of the trials.

### 3.5. Vitamin D Supplementation and Cardiovascular Disease-Related Outcomes

Of the 23 studies included in this review, 4 trials evaluated the effect of vitamin D therapy on surrogate measures of CV disease including left ventricular mass and function, abdominal aortic calcification (AAC), and pulse wave velocity (PWV) [29,31,33,35]. Four trials reported clinical CV events [22,27,28,29]. Two trials evaluated the response of brain natriuretic peptide (BNP), a biomarker of congestive heart failure, to vitamin D treatment [35,38]. These results are summarized in Table 5. All trials were placebo-controlled with the exception of one open-label study where standard therapy was the control arm [22]. Follow-up ranged from six months [28] to two years [22]. Overall, no treatment difference was observed in left ventricular mass or function, PWV or AAC [29,31,33,35]. In fact, PWV and AAC increased over time in both vitamin D-treated and untreated control participants [31,33,35]. Vitamin D treatment did not modify 24 h blood pressure, nor did it change levels of BNP [35,38]. There were significantly fewer deaths related to CV disease in the vitamin D-treated patients in the study by Brimble et al.; however, this was an exploratory analysis where the overall event rate was small and follow-up extended beyond the treatment period [29]. In the largest trial that included 284 patients and 24 months of follow-up, no difference was observed in CV death, nonfatal MI, nonfatal CVA, fatal MI, or fatal stroke [22]. The second largest study of 276 participants did not report any difference in the rate of hospitalization for CV disease over the 6-month treatment period [28].

### 3.6. Vitamin D Supplementation and Inflammation

Eight trials reported changes in *C*-reactive protein (CRP), a clinically used biomarker of inflammation (Table 6) [15,28,30,32,33,34,35,37]. One study demonstrated a significant between-group difference in CRP favoring vitamin D treatment [34]. However, this was a small 12-week study that randomized 55 patients but only included 38 participants in the final analysis. The much larger study by Wang et al. randomized 746 patients on hemodialysis with elevated depressive scores to 50,000 IU/week of cholecalciferol versus placebo and reported no difference in hs-CRP over the duration of the 12-month trial [15]. The second largest trial conducted by Miskulin et al. found a significant within-group decrease in hs-CRP in the group treated with ergocalciferol (*p* = 0.02), but no difference was observed in the change between the two groups [28]. The remaining trials randomized small numbers of patients and found no difference in CRP with vitamin D treatment [30,32,33,35,37].

### 3.7. Vitamin D Supplementation and Musculoskeletal Outcomes

Two trials reported outcomes related to muscle strength [33,40], and one trial evaluated changes in serum levels of testosterone [41]. In two studies, the change in grip strength was the primary outcome of the study. Neither study demonstrated any treatment benefit over 6 months and 12 months, respectively. Hewitt also assessed functional capacity and timed walking with no differences found between treatment arms [33]. Baseline 25(OH)D levels correlated with the distance walked in 6 min, but not muscle strength; however, no changes were detected over the 6-month supplementation period. One trial was designed to determine whether cholecalciferol treatment increases serum testosterone levels in patients with ESKD. Despite normalization of serum 25(OH)D levels, testosterone levels did not change [41]. Further, there was no correlation between testosterone and 25(OH)D levels at baseline or at the end of the study, suggesting that testosterone levels were independent from vitamin D status. Results were similar in males and females. No difference was reported in any study reporting fracture outcomes or falls. One small trial, published as an abstract only, reported a benefit in BMD preservation in favor of vitamin D [21]. In Zheng et al., BMD increased in the vitamin D and placebo groups; however, both arms received co-treatment with a vitamin D analog and a calcimimetic [25]. The results of these trials are summarized in Table 7.

### 3.8. Vitamin D Supplementation and Anemia, Pruritis, and Arteriovenous Fistula Maturation

One trial was designed, and thus powered, to determine whether vitamin D supplementation modified erythropoetin (EPO) dosage [28]. The overall results of this trial were negative. Two smaller trials, including fewer than 100 participants in each, also reported a change in EPO dose. The study conducted by Mehorotra et al., reported in abstract form only, reported a significant decrease in EPO dosage in the treatment group [20]. Naini et al. reported no significant effect of treatment on EPO dose [36]. One trial measured change in pruritus symptoms over 12 weeks. Pruritus severity was measured using a survey and corresponding score. In both the treatment and placebo groups, there was a decrease in severity of itch over the time course of the study and there was no significant effect of treatment [39]. Wasse et al. measured the patency success of created hemodialysis access (arteriovenous fistula (AVF) or arteriovenous graft (AVG)) in patients randomized to either vitamin D or matching placebo [24]. After 6 months, the percentage of patients with successful cannulation of their AVF or AVG was not significantly different between the two groups [24]. These results are summarized in Table 8.

### 3.9. Vitamin D and Metabolic/Nutritional Measures

Five trials included a measure of nutritional or metabolic health as an outcome. The results of these outcomes are summarized in Supplemental Table S1. Serum albumin was measured in five of the trials. In each of these trials [15,29,30,32,38] there was no significant change in albumin between placebo or treatment groups, although there was a trend towards higher levels of albumin and prealbumin in the vitamin D-treated group in the largest study [15].

Khajehdehi et al. also measured lipids in patients randomized to either vitamin D, vitamin C, or vitamin E with each group compared to placebo [26]. They found that, compared to the placebo group, participants in the vitamin D group had significantly lower triglycerides and a lower triglyceride to HDL-c ratio at study end. There were no differences at study exit from baseline in either the vitamin D or placebo group in LDL-c/HDL-c ratio, cholesterol/HDL-c ratio, cholesterol and LDL-c, and HDL-c [26].

### 3.10. Vitamin D Supplementation and Well-Being

Ayub et al. used a participant-reported survey to evaluate chronic pain. They found a significant reduction in reported pain in both the treatment and placebo groups [30]. Hewwit et al. used the Kidney Disease Quality of Life (KDQOL)-36 to assess kidney-disease-related quality of life (Supplemental Table S2) [33]. Baseline 25(OH)D levels did not correlate with HRQOL at baseline, and there was no difference between treated participants and controls after 6 months [33]. Singer et al. used the KDQOL—Short Form (KDQOL-SF) to assess quality of life related to kidney-failure-specific symptoms [40]. After 12 months of cholecalciferol treatment, there was no difference in the KDQOL-SF scores between the treated and placebo groups. Re-analysis restricted only to those with more severe baseline vitamin D deficiency (25(OH)D < 27.5 nmol/L) did not change these results. Further, no differences were observed in any of the KDQOL-SF domains [40]. Only one trial measured psychological health as an outcome. Wang et al. used the Chinese version of Beck’s Depressive Inventory II (BDI-II) as a measure of depressive symptoms in hemodialysis patients [15]. The BDI-II evaluates 21 self-reported items on a scale of 0–3, giving a total possible score of 63. Scores over 11 reflect the presence of depressive symptoms [42]. Participants in the study required a score of 16 or higher to be eligible. At baseline, there were no significant differences between the vitamin D and placebo groups. After 12 months, BDI-II scores were significantly lower compared to baseline in both the vitamin D and placebo groups, and there was no significant difference in the mean change in score between the two groups [15]. In a sub-analysis, participants were stratified by type of depression. In participants with a diagnosis of vascular depression at baseline, there was a significantly larger mean decrease in BDI-II scores (−5.5 ± 0.6 vs. −1.4 ± 0.3, *p* = 0.047) in patients treated with vitamin D (n = 150) compared to patients taking the placebo (n = 160) [15]. In participants with diagnosed major depressive disorders (MDDs) at baseline, there was no significant difference in mean change in BDI-II score by treatment group.

### 3.11. Vitamin D and Changes in 25(OH)D, Hypercalcemia, and Vitamin D Toxicity

All studies but one [26] included in this review measured the response of serum 25(OH)D levels to vitamin D treatment (Supplemental Table S3). Overall, a significant treatment effect on the increase in 25(OH)D was observed. In only one trial, a substantial proportion of study participants did not reach sufficient vitamin D levels at study end [22]. We determined the frequency of hypercalcemia and vitamin D toxicity (25(OH)D > 250 nmol/L). Vitamin D toxicity was only specifically addressed in three trials, and a vitamin D level > 250 nmol/L was reported in three patients (one treated patient and two controls). Hypercalcemia was specifically mentioned in 15 trials but occurred infrequently.

## 4. Discussion

To date, this is the largest systematic review examining native vitamin D therapy in patients with ESKD requiring dialysis that focuses on the potential pleiotropic benefits of vitamin D beyond those related to mineral homeostasis. Our search criteria started in 1947, yet 20 of these trials were published within the past 10 years, indicating a renewed interest in native vitamin D in this patient population. Overall trends in the data show limited effectiveness of vitamin D therapy on a variety of clinical outcomes. Previous systematic reviews confirmed that treatment with native vitamin D corrects vitamin D deficiency in patients with ESKD [43,44]. The results of this review suggest that repletion of vitamin D does not appear to parallel subsequent changes in outcomes beyond measures of vitamin D sufficiency. However, the majority of trials (19 of 23) randomized 100 or fewer participants, indicating that, overall, the studies were quite small with limited power to address the outcomes. Further, many trials were of low-to-moderate quality. From the two largest, and unbiased, trials, we find no evidence that vitamin D replacement decreases erythropoietin usage or improves depressive symptoms or levels of CRP in patients with ESKD [15,28].

In a meta-analysis of 50 randomized trials that included almost 75,000 participants without CKD, vitamin D supplementation was not associated with a reduced risk of all-cause mortality risk [45]. Whether these results can be applied to a population with ESKD, where vitamin D metabolism is dysregulated and vitamin D deficiency is frequently encountered, is unknown. In a meta-analysis of observational studies, vitamin D treatment was associated with survival in patients with earlier stages of CKD [46]. The small sample sizes of the randomized studies included in our review would suggest that none of these trials were powered adequately to detect an impact of vitamin D supplementation on overall survival, should one exist. The nephrology community awaits the results of the simplified trial. This large, pragmatic trial of over 4000 dialysis patients in the United Kingdom is a prospective, randomized, open-label blinded-endpoint superiority trial comparing cholecalciferol versus standard care in patients on dialysis [47]. The primary outcome for the simplified trial is patient survival.

CV disease is much more common in patients with ESKD than in the general population; however, this prevalence is not explained by traditional CV risk factors [48]. Other factors contribute, including vascular calcification related to dysregulated mineral metabolism and inflammation [48]. Vitamin D receptors are expressed widely, and CYP27B1 has been found in cardiac tissue, vascular smooth muscle cells, and endothelium [10]. Stimulation of vitamin D receptors in the myocardium can prevent cardiac hypertrophy and decrease secretion of BNP, a biomarker used clinically to evaluate congestive heart failure [49]. In the general population, vitamin D supplementation does not appear to reduce CV events [50,51]. However, the majority of these trials were primary prevention trials in populations where vitamin D deficiency is uncommon, and the CV event rate was typically a secondary outcome. The duration of follow-up may not have been sufficient to capture events related to a chronic disease in healthier people. In our review, a surrogate outcome of CV disease was the primary outcome in four trials. In prospective and non-randomized studies of patients receiving dialysis, vitamin D treatment decreased left ventricular mass and reduced levels of BNP [52,53]. However, the two randomized and controlled studies that included a measurement of left ventricular mass showed no difference between vitamin D treatment versus placebo [29,35]. Similarly, we found no treatment effect of vitamin D on BNP, PWV, abdominal aorta calcification, or 24 h BP [31,33,35,38]. Taken together, there is no available evidence from these relatively small trials to suggest that vitamin D alters CV structure and/or function in patients receiving dialysis.

Clinical CV event rates were assessed in four studies. In the largest trial of 276 patients receiving HD that compared 6 months of ergocalciferol supplementation to placebo, there was a trend toward reduced hospitalization for a CV cause, favoring those receiving vitamin D [28]. However, estimates would be expected to be imprecise given the sample size, and the study was not powered to detect this outcome. In a sub-group analysis, there was no difference by subgroups of baseline 25(OH)D concentrations. In a much smaller trial of PD patients, there was a significant decrease in death from CV disease in patients treated with vitamin D, but caution is required when interpreting this due to the overall very small number of events and extension of follow-up beyond the treatment period [29].

Patients requiring hemodialysis are chronically inflamed, and its presence is associated with poor outcomes including CV events [54]. In this population, low serum levels of 25(OH)D have been associated with elevated levels of CRP and IL-6 [55]. Vitamin D has been identified as a potential modifier of inflammation where it has been proposed that 1.25(OH)_2_D, synthesized in monocytes, may inhibit the production of pro-inflammatory cytokines [56]. Recent meta-analyses have demonstrated that vitamin D supplementation improved levels of CRP in the general population [57], in patients with diabetes [58], as well as in patients with diabetic kidney disease [59]. In our review, two studies demonstrated a significant difference in CRP levels with supplementation; however, there was significant loss to follow-up in both studies [30,34]. The remaining randomized trials did not show any difference in CRP levels despite substantial improvement in 25(OH)D levels, a longer duration of follow-up (6–12 months), and much larger sample sizes [15,28]. Overall, there is minimal evidence from randomized trials to support a role for vitamin D supplementation in reducing levels of CRP in patients receiving dialysis.

Low serum testosterone levels are associated with mortality in male patients receiving HD, and in early stages of CKD, testosterone levels have been linked to muscle strength [60,61]. Hypogonadism has been linked to vitamin D deficiency in several patient cohorts, but whether vitamin D replacement modifies testosterone levels in patients receiving HD is not known [62,63]. The one trial that evaluated testosterone levels did not show any change over 12 weeks; however, the sample size was very small [41]. Neither trial that evaluated measures of muscle strength demonstrated any benefit of vitamin D [33,40]. Although these trials do not support the use of vitamin D in hemodialysis patients for muscle and strength outcomes, the sample sizes are small, and the duration of follow-up may not be long enough to find a significant effect of treatment, should one exist.

Osteoporosis is a key component of bone disease in this population where fractures occur frequently. Vitamin D supplementation has been shown to decrease the incidence of bone fracture in people above the age of 60 but not in younger individuals [50,64]. The impact of vitamin D on bone mineral density is not proven in the general population but may benefit those individuals above the age of 50 [50,65]. In our review, BMD results were presented in one abstract, one uncontrolled study of 19 participants, and a placebo-controlled study of 60 participants who were also receiving cinacalcet and a vitamin D analog [21,23,25]. No conclusions can be reasonably drawn from these limited data, and specifically designed RCTs would be necessary to confirm or refute the observational data from other elderly patient groups.

HRQOL is significantly reduced in patients with ESKD on HD and has been associated with numerous adverse outcomes including hospitalization and mortality [66]. Low levels of 25(OH)D have been associated with reduced HRQOL in patients receiving dialysis [67]. In the trial conducted by Hewitt et al., baseline HRQOL scores were not associated with 25(OH)D levels, and following six months of vitamin D supplementation, there were no between-group differences in HRQOL domains [33]. In the 12-month trial, supplementation resulted in substantial differences in achieved 25(OH)D levels between groups but did not translate into differences in HRQOL measures [40]. One study of 746 patients randomized hemodialysis patients with depression [15]. Vascular depression describes depressive disorders in later life in patients with either clinical or imaging evidence of CV disease. In the sub-group of patients with vascular depression, there was some benefit in favor of cholecalciferol, and the authors attributed this as being dependent on improvements in unmeasured CV factors [15].

A number of trials evaluated the impact of vitamin D on outcomes that are unique to the ESKD population. Many patients with ESKD experience pruritis, which can have significant impact on sleep quality and mood [68,69]. Ultraviolet light, which increases the cutaneous production of calcitriol via vitamin D precursors, has been shown to improve symptoms in patients with refractory pruritis [70,71]. One trial sought to determine whether vitamin D might have anti-pruritic effects. However, no significant difference in pruritis severity scores between the placebo and treated groups was noted at any time point during the 12-week study [39], including in those supplemented patients that converted from vitamin D insufficiency to repletion. Both groups experienced a reduction in pruritis scores, suggesting either abatement of itching symptoms over time or regression to the mean [39]. Although increasing 1.25(OH)_2_D production in the skin is an attractive hypothesis to modify cutaneous immunity and down-regulate cutaneous inflammation, this small trial did not support this.

Given a putative role for vitamin D in suppressing neointimal hyperplasia, it was hypothesized that vitamin D supplementation might improve arteriovenous fistula (AVF) outcomes. This pilot study was conducted to determine the feasibility of a trial to determine whether high-dose cholecalciferol, compared to placebo, modified the maturation of AVFs [24]. At follow-up, there was no difference in successful cannulation between cholecalciferol- and placebo-treated groups [24]. Conclusive results might require a longer period of treatment prior to AVF creation, a larger sample size, and a study population at risk for non-maturation.

Anemia has been associated with poor quality of life and increased CV complications and mortality in patients with ESKD [72]. The majority of patients receive erythropoietin for anemia correction; however, a fraction of these patients are hypo-responsive and require large doses. Vitamin D-mediated suppression of inflammatory cytokines is one postulated mechanism by which vitamin D may promote erythropoeisis [73]. One randomized trial, adequately powered to evaluate whether 6 months of vitamin D supplementation reduced erythropoietin dose requirements in patients receiving HD, was negative [28]. The authors also conducted a sensitivity analysis that did not demonstrate any effect on erythropoetin dose in participants who achieved vitamin D sufficiency. Two much smaller trials suggested that administration of vitamin D might reduce erythropoietin doses; however, these trials studied far fewer participants over a shorter duration and performed poorly on several measures of bias [20,36]. The study by Miskulin et al. was adequately powered to address the primary outcome of epoetin usage; therefore, we determine that vitamin D supplementation does not play a significant role in the management of anemia in ESKD [28].

The results of this systematic review confirm that nutritional vitamin D insufficiency and deficiency can be safely corrected in patients with ESKD. It is therefore unlikely that the mostly negative results were on the basis of not achieving adequate vitamin D levels or that different results would have been obtained if higher doses were used. Overall, hypercalcemia was infrequent and typically not higher in patients randomized to vitamin D. Only three studies [22,31,40] specifically addressed the upper limit of vitamin D, and in these studies only one patient exceeded the threshold. Although the results do not indicate a vitamin D-treatment benefit on a variety of pleiotropic outcomes, it is unlikely that this was based on unsuccessful correction of vitamin D deficiency. Further, in those studies that conducted a sensitivity analysis based on adequate correction of vitamin D, the negative conclusions remained unchanged. Limitations of this systematic review relate to overall study quality and the small sample sizes with limited power to address the outcomes. Due to the underlying heterogeneity in terms of interventions and captured outcomes, the data are not amenable to meta-analysis. From the majority of trials we can exclude large effects of vitamin D treatment on the various outcomes; however, smaller effect sizes may still be possible. This review is the first systematic review in the literature that examines randomized trials of vitamin D treatment and pleiotropic outcomes in patients receiving dialysis where a high prevalence of vitamin D deficiency exists. There is a lack of adequately powered randomized trial evidence to support a beneficial role for vitamin D in outcomes relating to its potential pleiotropic effects. At present, it is not clear from clinical trials conducted in humans with ESKD that a causal pathway exists between 25(OH)D and clinical outcomes that operates through correction of vitamin D deficiency and is responsive to vitamin D treatment.

## Figures and Tables

**Table 1:** PICOS (population, intervention, comparison, outcomes, and settings) criteria for the inclusion of studies evaluating the effects of vitamin D supplementation on clinical outcomes.

Parameter	Inclusion Criteria
Population	End-stage kidney disease requiring dialysis treatment
Intervention	Native vitamin D
Comparison	Non-exposed control group
Outcomes	Any clinical or measured outcome
Settings	Randomized trials

**Table 2:** Characteristics of included studies.

Author Year Country # Centers	Population	Follow-Up (mo)	Intervention	Control	Randomized (n)		Included in Final Analysis (n)		Primary Outcome of the Trial	Clinical Outcome Measures Included In Review	Age (Mean (SD))	
					VD	Control	VD	Control			VD	Control
Ambrus ^#^ 2003 * Hungary 1 [21]	HD	12	D3, 3000–9000 IU weekly	Standard care	45	45	NR	NR	Mineral metabolism and VD status	Femoral neck BMD (g/cm^2^)	58 ± 15	56 ± 15
Ayub 2022 Pakistan 1 [30]	HD 25D < 75 nmol/L	2	D3, 50,000 IU weekly in 25D < 37 nmol/L, 10,000 IU weekly in 25D 40–75 nmol/L	Placebo	35	35			Biomarkers of mineral metabolism	CRP, VAS for pain, albumin, Hb	49.5 ± 10.1	46.9 ± 14
Bhan 2015 United States 3 [27]	HD 25D < 80 nmol/L	4	D2, 50,000 IU weekly	Placebo	36	36	36	36	25D levels	All-cause mortality, all-cause and cause-specific hospitalizations	53 ± 17	59 ± 17
			D2, 50,000 IU monthly		33		33				58 ± 16	
Brimble 2022 Canada 6 [29]	PD	12	D3, 50,000 IU weekly for 8 weeks, followed by 10,000 IU weekly for 44 weeks and BIA guided care	Placebo and BIA guided care	19	13	15	9	Change in LV mass (cardiac MRI)	Composite of death, nonfatal CV event, transfer to HD, fractures	61.5 ± 13.3	61.7 ± 11.9
			D3, 50,000 IU weekly for 8 weeks, followed by 10,000 IU weekly for 44 weeks and standard care	Placebo and standard care	15	18	14	15				
Delanaye 2013 Belgium 3 [31]	HD 25D < 75 nmol/L	12	D3, 25,000 IU bi-weekly	Placebo	22	21	16	14	25D levels	AAC score, PVW	75 ± 9	73 ± 12
Gregorio, 2021 * Brazil 1 [32]	HD 25D < 75 nmol/L	6	D3, 50,000 IU weekly. After 3 mo, patients with sufficient 25(OH)D received 50,000 IU monthly	Placebo	18	14	12	11	Inflammation (in vitro assays, CRP)	Hb, albumin	59.0 ** [51.8–60.3]	55.5 ** [50.5–65.23]
Hewitt 2013 Australia NR [33]	HD 25D < 60 nmol/L	6	D3, 50,000 IU weekly for 8 weeks, followed by monthly for 4 months	Placebo	30	30	29	27	Muscle strength	Functional capacity, HRQOL, PWV, fractures	60 ** [53, 71]	67 ** (54, 72)
Khajehdehi, 2000 Iran NR [26]	HD	3	D3, 50,000 IU daily	Placebo	21	21	15	14	Serum values of triglyceride, cholesterol, LDL-c, and HDL-c		Median 31.4 years	
Mehrotra ^#^ 2013 * United States NR [20]	HD 25D < 62 nmol/L	6	D3, 50,000 IU weekly	Standard care	51	28	NR	NR	EPO dose	Hb	NR	NR
Meireles 2016 Brazil 1 [34]	HD, PD 25D < 50 nmol/L	3	D3, 50,000 IU twice weekly	Placebo	28	27	20	18	Expression of VDR in monocytes	CRP	55.5 ± 14.2	56.5 ± 12.9
Mieczkowski 2014 Poland 1 [23]	HD 25D < 50 nmol/L	12	D3, 2000 IU 3 times/week	Standard care	8	11	NR	NR	Vitamin D levels	BMD	63 (52–79)	46 (29–79)
Miskulin 2016 United States 12 [28]	HD 25D ≤ 75 nmol/L	6	D2, 50,000 IU weekly or weekly for 3 months then monthly (depending on baseline 25D status)	Placebo	137	139	122	130	EPO dose	CRP cardiovascular hospitalizations, falls, fractures	61.0 ± 13.3	60.8 ± 13.9
Morrone, 2021 * Italy 28 [22]	HD 25D < 75 nmol/L	24	Calcifediol, 40 mcg thrice weekly	Standard care	143	141	143	141	Nonfatal MI, nonfatal stroke, and death from any cause (except trauma)	Cardiovascular and non-cardiovascular mortality, fatal MI, fatal stroke	67.1 ± 13.2	65.1 ± 12.6
Mose 2014 Denmark 1 [35]	HD, PD	6	D3, 3000 IU daily	Placebo	32	32	25	25	*p*-BNP	24-h BP, PWV, and CRP LVMI LVEF	68 ± 9	67 ± 13
Naini 2015 Iran 1 [36]	HD 25D < 75 nmol/L Hb < 110 gLl	4	VD not specified, 50,000 IU weekly for 12 weeks and then every three weeks until participants reached 650,000 IU	Placebo	32	32	32	32	Hb, EPO dose		60 ± 19	62 ± 21
Seibert 2013 Germany 1 [37]	HD 25D < 80 nmol/l	3	D3, 20,000 IU twice weekly to once monthly depending on baseline VD status	Placebo	19	19	15	18	Number of CD14+ and CD16+ cells per mL of blood	CRP	66.9 ± 10.8	67.4 ± 9.8
Seirafian 2014 Iran 1 [38]	PD, 25D < 75 nmol/L	3	VD, 50,000 IU weekly for 12 weeks if 25D level was <25 nmol/L and for 8 weeks if 25–75 nmol/L	Placebo	49	40	46	38	BNP	Albumin	55.1 ± 17.4	54.6 ± 13.5
Shirazian 2013 United States 1 [39]	HD, with excessive pruritis	3	D3, 50,000 IU weekly	Placebo	25	25	25	25	Pruritus severity		66.1 ± 14.7	66.2 ± 13.7
Singer 2019 Australia 1 [40]	PD, HD 25D < 50 nmol/L	12	D3, 50,000 IU weekly	Placebo	36	32	29	26	KDQOL-SF, grip strength	Blood pressure, cardiac ischemia	59.5 ± 15.6	63.8 ± 14.2
Ulrich 2021 Germany 1 [41]	HD	3	D3, 800 IU daily	Placebo	19	19	15	18	Testosterone		66.9 ± 10.8	67.4 ± 9.8
Wang 2016 China 3 [15]	HD, PD 25D 37–80 nmol/L	12	D3, 50,000 IU weekly	Placebo	373	373	362	364	BDI-II	MDDs, vascular depression CRP	NR	NR
Wasse, 2014 United States 1 [24]	HD preparing to receive AVF within 4 weeks	0.75	D3, 200,000 IU weekly	Placebo	25	27	20	24	25D levels	Arteriovenous access maturation at 6 months	49.9 ± 10.9	52.1 ± 14.9
Zheng *, 2018 Taiwan [25]	HD, with severe SHPT	6	D3, 500 IU daily; Cinacalcet, 30 mcg daily; calcitriol (unique dose)	Placebo, Cinacalcet 30 mcg daily, Calcitriol (unique dose)	30	30	27	28	Serum iPTH < 300 pg/mL	10% improvement in femoral neck BMD	66.2 ± 12.8	65.6 ± 13.4

^#^ Abstract only; * open-label/single-blinded trials; ** median age [interquartile range]; AAC: abdominal aortic calcification; BDI-II: Beck’s Depression Index-II; BMD: bone mineral density; BNP: brain natriuretic peptide; D2: ergocalciferol; D3: cholecalciferol; DBP: diastolic blood pressure; CRP: *C*-reactive protein; EPO: erythropoietin; Hb: hemoglobin; HD: hemodialysis; HDL: high-density lipoprotein; HRQOL: health-related quality of life; iPTH: intact parathyroid hormone; IU: international units; KDQOL-SF: kidney disease quality of life—short form; LDL: low-density lipoprotein; LVEF: left ventricular ejection fraction; LVM: left ventricular mass; LVMI: left ventricular mass index; MDDS: major depressive disorders; MI: myocardial infarction; NR: not reported; PD: peritoneal dialysis; PWV: pulse wave velocity; SBP: systolic blood pressure; SHPT: secondary hyperparathyroidism; VAS: visual analog scale; VD: vitamin D; VDR: vitamin D receptor; 25D:25-hydroxyvitamin D level.

**Table 3:** Bias assessment of included studies.

Author	Randomization Process	Effect of Assignment to Intervention	Effect of Adhering to Intervention	Missing Outcome Data	Measurement of Outcomes	Selection of the Reported Results
Ambrus ^#^, 2003 [21]	Some concerns	High	High	High	Some concerns	Some concerns
Ayub, 2022 [30]	Some concerns	High	High	High	Low	Some concerns
Bhan, 2015 [27]	Low	Low	Low	Low	Low	Low
Brimble, 2022 [29]	Low	Low	Low	Low	Low	Low
Delanaye, 2013 [31]	Low	Low	Low	Some concerns	Low	Low
Gregorio, 2021 [32]	Some concerns	Low	Low	High	Low	Some concerns
Hewitt, 2013 [33]	Low	Low	Low	Low	Low	Low
Khajehdehi, 2000 [26]	High risk	High	High	High	Low	Some concerns
Mehrotra ^#^, 2013 [20]	Some concerns	High	High	High	High	Some concerns
Meireles, 2016 [34]	Low	Low	Some concerns	High	Low	Low
Mieczkowski, 2014 [23]	Low	High	High	High	Some concerns	Some
Miskulin, 2016 [28]	Low	Low	Low	Low	Low	Low
Morrone, 2021 [22]	Low	Low	Some concerns	Low	Some concerns	Low
Mose, 2014 [35]	Low	Low	Low	Low	Low	Some concerns
Naini, 2015 [36]	Some concerns	Some concerns	High	High	Low	Low
Seibert, 2013 [37]	Low	Low	Low	Low	Low	Low
Seirafian, 2014 [38]	Some concerns	Low	Low	Low	Low	Low
Shirazian, 2013 [39]	Low	Low	Low	Low	Low	Some concerns
Singer, 2019 [40]	Low	Low	Low	low	Low	Low
Ulrich, 2021 [41]	Low	Some concerns	Some concerns	High	Low	Low
Wang, 2016 [15]	Low	Low	Some	Low	Low	Some concerns
Wasse, 2014 [24]	Some concerns	Low	Low	low	Low	Low
Zheng, 2018 [25]	High	Low	Low	Low	High	High

^#^ Abstract only.

**Table 4:** Summary of mortality and hospitalization outcomes.

Author, Year	Length of Follow-Up (mo)	Study Arms	Randomized (n)	Lost to Follow-Up (n)	Included in Final Analysis (n)	Outcome Details	Outcome Measurements	*p* Value *
							End of Follow-Up	
**All-cause mortality**								
Bhan, 2015 [27]	4 ^1^	D2 weekly	36	0	36	All-cause mortality (n,%)	3 (8.3%)	0.08
		D2 monthly	33	0	33		0 (0%)	
		Placebo	36	0	36		5 (13.9%)	
Brimble, 2022 [29]	12 ^2^	D3, BIA, or standard care	34	0	34	All-cause mortality (n,%)	4 (12%)	0.004
		Placebo, BIA, or standard care	31	0	31		12 (39%)	
Morrone, 2021 [22]	24	Calcifediol	143	26	143	Death (any cause, excluding trauma or accidental) (n,%)	33 (23.1%)	HR (95% CI): 1.11 (0.67–1.83) ns
		Standard care	141	28	141		28 (19.9%)	
Singer, 2019 [40]	12	D3	36	7	29	Death (n,%)	1 (3%)	0.54
		Placebo	32	6	26		0 (0%)	
**Hospitalizations and adverse events (excluding cardiovascular events)**								
Bahn, 2015 [27]	4 ^1^	D2 weekly	36	0	36	Hospitalization (n,%)	14 (38.9%)	0.89
		D2 monthly	33	0	33		11 (33.3%)	
		Placebo	36	0	36		13 (36.1%)	
		D2 weekly	36	0	36	Infections (n,%)	11 (30.6%)	0.56
		D2 monthly	33	0	33		11 (33.3%)	
		Placebo	36	0	36		8 (22.2%)	
		D2 weekly	36	0	36	Respiratory events (n,%)	3 (8.3%)	0.92
		D2 monthly	33	0	33		2 (6.1%)	
		Placebo	36	0	36		3 (8.3%)	
		D2 weekly	36	0	36	Adverse events (n,%)	33 (91.7%)	0.22
		D2 monthly	33	0	33		26 (78.8%)	
		Placebo	36	0	36		28 (77.8%)	
Brimble, 2022 [29]	12 ^2^	D3	34	0	34	Limb amputation (n,%)	1 (3%)	0.2
		Placebo	31	0	31		2 (7%)	
		D3	34	0	34	Hospitalizations per patient (n,%)	2 (1–3)	0.7
		Placebo	31	0	31		2 (1–3)	
Miskulin, 2016 [28]	6	D2	137	15	122	All-cause hospitalization IRR	0.82 (0.60 to 1.12)	0.20
		Placebo	139	9	130		1.00 (ref)	
		D2	137	15	122	Infection-related hospitalization IRR	1.03 (0.50 to 2.10)	0.95
		Placebo	139	9	130		1.00 (ref)	
Morrone, 2021 [22]	24	Calcifediol	143	26	143	Hospitalization (n,%)	21 (14.7%)	0.51
		Standard care	141	28	141		16 (11.3%)	
		Calcifediol	143	26	143	Serious adverse events (n,%)	45 (31.5%)	0.48
		Standard care	141	28	141		39 (27.0%)	
		Calcifediol	143	26	143	Non-CV death (n,%)	24 (16.8)	HR (95% CI): 1.13 (0.63–2.04)
		Standard care	141	28	141		20 (14.2)	
Singer, 2019 [40]	12	D3	36	7	29	Hospital admission (n,%)	22 (61%)	0.63
		placebo	32	6	26		17 (53%)	

CV: cardiovascular; HR: hazard ratio; IRR: incidence rate ratio; ns: not significant. * *p* value represents the significance level of the reported treatment effect between groups; ^1^ treatment was 4 months and follow-up was 12 months; ^2^ treatment was 3 months and follow-up was >1 year.

**Table 5:** Vitamin D supplementation and cardiovascular outcomes.

Author, Year	Length of Follow-Up (Mo)	Study Arms	Randomized (n)	Lost to Follow-Up (n)	Included in Final Analysis (n)	Outcome Details	Outcome Measurements		*p* Value *
							Baseline	End of Study	
**Surrogate cardiovascular endpoints**									
Brimble, 2022 [29]	12	D3	34	5	29	LVM (g) ^1^	144.2 ± 50.2	134.3 ± 47.1	0.6
		Placebo	31	7	24		142.8 ± 52.3	136.3 ± 52.7	
		D3	34	5	29	LVMI (g/m^2^)	76.0 ± 25.4	69.1 ± 21.6	0.4
		Placebo	31	7	24		73.2 ±23.0	70.6 ± 24.5	
		D3	34	5	29	LVEF (%)	58.5 ± 8.6	56.5 ± 10.0	0.7
		Placebo	31	7	24		56.5 ± 10.3	56.5 ± 8.7	
Delayne, 2013 [31]	12	D3	22	6	16	AAC score	8 ± 5	10 ± 6	0.89
		Placebo	21	7	14		8 ± 8	1 ± 7	
Hewitt, 2013 [33]	6	D3	30	9	21	PWV (m/s)	NR	9.3 ± 3.3	0.76
		Placebo	30	6	24		NR	10.5 ± 2.8	
Mose, 2014 [35]	6	D3	32	7	25	24 h SBP (mmHg)	135 ± 18	130 ± 14	0.511
		Placebo	32	7	25		136 ± 22	127 ± 23	
		D3	32	7	25	24 h DBP (mmHg)	73 ± 9	71 ± 8	0.451
		Placebo	32	7	25		73 ± 10	69 ± 10	
		D3	32	10	22	PWV (m/s)	9.7 ± 2.5	10.5 ± 4.0	0.269
		Placebo	32	13	19		10.0 ± 2.0	10.1 ±2.5	
		D3	32	10	22	LVEF % ^2^	53 ± 14	56 ± 12	0.515
		Placebo	32	8	24		52 ± 14	52 ± 17	
		D3	32	10	22	LVMI (g/m^2^)	123 ± 34	127 ± 50	0.397
		Placebo	32	8	24		116 ± 36	111 ± 39	
**Cardiovascular events (death, MI, stroke)**									
Bahn, 2015 [27]	4 ^3^	D2 weekly	36	0	36	CV events (n, %)	6 (16.7%)		0.31
		D2 monthly	33	0	33		2 (6.1%)		
		Placebo	36	0	36		3 (8.3%)		
Brimble, 2022 [29]	12	D3	34	0	34	CV events (n, %)	5 (15%)		0.4
		Placebo	31	0	31		7 (23%)		
		D3	34	0	34	Deaths from CV cause (n,%)	1 (3%)		0.03
		Placebo	31	0	31		6 (19%)		
		Placebo	31	0	31		2 (7%)		
Miskulin, 2016 [28]	6	D2	137	15	122	CV disease hospitalization IRR	0.60 (0.33–1.09)		0.1
		Placebo	139	9	130		1.00 (ref)		
Morrone, 2021 [22]	24	Calcifediol	143	26	143	Cardiovascular Death (n,%)	9 (6.3%)		HR (95 CI): 1.06 (0.41–2.74)
		Standard care	141	28	141		8 (5.7%)		
		Standard care	141	28	141		0 (0%)		
**Biomarker outcomes (BNP)**									
Mose, 2014 [35]	6	D3	32	7	25	BNP (pmol/L)	61 (26, 378)	95 (35, 363)	0.82
		Placebo	32	7	25		81 (24, 186)	50 (30, 265)	
		Placebo	32	7	25		10 (4, 19)	8 (6, 23)	
Seirafian, 2014 [38]	3	VD	49	3	46	Pro-BNP (pg/mL)	8951 ± 1631	895.9 ± 779.6	0.50 **
		Placebo	40	2	38		7933 ± 1492	736.7 ± 797.9	0.52 **

AAC: abdominal aortic calcification score; BNP: brain natriuretic peptide; CV: cardiovascular; DBP: diastolic blood pressure; IRR: incidence rate ratio; LVEF: left ventricular ejection fraction; LVM: left ventricular mass; LVMI: left ventricular mass index; MI: myocardial infarction; SBP: systolic blood pressure. * *p* value represents the significance level of the reported treatment effect between groups; ** *p* value represents treatment effect within groups from baseline. ^1^ Measured by magnetic resonance imaging; ^2^ measured by echocardiogram; ^3^ subsequent participant follow-up for CV events was 12 months.

**Table 6:** Vitamin D supplementation and CRP outcomes.

Author, Year	Length of Follow-Up (mo)	Study Arms	Randomized (n)	Lost to Follow-Up (n)	Included in Final Analysis (n)	Outcome Details	Outcome Measurements		*p* Value *
							Baseline	End of Follow-Up	
Ayub, 2022 [30]	2	D3	35			CRP (mg/dL)	6.8 ± 4.2	5.0 ± 3.7	0.005
		Placebo	35				7.9 ± 4.5	7.5 ± 3.8	
Gregiorio, 2021 [32]	6	D3	18	6	12	hs-CRP (mg/dL)	0.44 [0.25, 1.2]	0.57 [0.17, 2.0]	ns
		Placebo	14	3	11		0.25 [0.1, 1.1]	0.44 [0.2, 1.9]	
Hewitt, 2013 [33]	6	D3	30	9	21	CRP (mg/L)	9 [5, 17]	NR	ns
		Placebo	30	6	24		10 [5, 20]	NR	
Miereles, 2016 [34]	3	D3	28	8	20	CRP (mg/dL)	0.50 [0.1, 1.3]	0.28 [0.1, 0.6]	<0.05 **
		Placebo	27	9	18		0.57 [0.2, 1.7]	0.48 [0.2, 1.7]	
Miskulin, 2016 [28]	6	D2	137	15	122	hs-CRP (mg/L)	5.1 [1.8, 10.3]	5.9 * [2.0, 14.5]	0.22
		Placebo	139	9	130		3.8 [1.5, 12.0]	4.4 [1.7, 10.9]	
Mose, 2014 [35]	6	D3	32	7	25	CRP (mg/L)	3.4 [1.1, 13.3]	3.9 [1.1, 11.3]	0.24
		Placebo	32	7	25		4.5 [1.7, 11.7]	2.5 [1.6, 13.9]	
Seibert, 2013 [37]	3	D3	19	4	15	CRP (mg/L)	4.8 [0.6–33.2]	7.5 [0.6–36.9]	ns
		Placebo	19	1	18		5.6 [0.8–19.4]	4.2 [0.6–14.5]	ns
Wang, 2016 [15]	12	D3	362	0	362	hs-CRP (mg/L)	9.1 ± 3.3	8.4 ± 3.1	0.48
		Placebo	364	0	364		10.5 ± 2.6	10.3 ± 3.6	

CRP: *C*-reactive protein; Hs-CRP: high-sensitivity *C*-reactive protein; NR: not reported; ns: not significant. * *p* value represents the significance level of the reported treatment effect between groups; ** *p* value represents treatment effect within groups from baseline (*p* < 0.05 in ergocalciferol patients at 6 months compared to baseline).

**Table 7:** Vitamin D supplementation and musculoskeletal outcomes.

Author, Year	Length of Follow-Up (mo)	Study Arms	Randomized (n)	Lost to Follow-Up (n)	Included in Final Analysis (n)	Outcome Details	Outcome Measurements		*p* Value *
							Baseline	Study Exit	
Miskulin, 2016 [28]	6	D2	137	15	122	Falls IRR	1.03 (0.56–1.88)		0.94
		Placebo	139	9	130		1.00 (ref)		
		D2	137	15	122	Fractures IRR	5.13 (0.60–43.88)		0.14
		Placebo	139	9	130		1.00 (ref)		
Brimble, 2022 [29]	12	D3	34	0	34	Fractures (n, %)	1 (3%)		0.5
		Placebo	31	0	31		2 (7%)		
Hewitt, 2013 [33]	6	D3	30	9	21	Fractures (n)	1		ns
		Placebo	30	6	24		0		
		D3	30	9	21	Grip strength (kg, 95 CI)	23 [19, 28]	24 [21, 28]	0.28
		Placebo	30	6	24		21 [17, 25]	21 [17, 24]	
Singer, 2019 [40]	12	D3	36	7	29	Grip strength (kg, IQR)	27.5 (22, 37.5]	26 [22, 38]	0.81
		Placebo	32	6	26		24 [20, 35.8]	27 [19, 39]	
Ambrus, 2003 [21]	12	VD	45	NR	NR	FN-BMD	0.75 ± 0.16	0.75 ± 0.16	<0.01
		Placebo	45	NR	NR		0.74 ± 0.17	0.70 ± 0.16	
Zheng, 2018 [25]	4	D3	30	3	27	FN-BMD (g/cm^2^)	0.57 ± 0.04	0.67 ± 0.07	<0.05 **
		Placebo	30	2	28		0.58 ± 0.05	0.62 ± 0.06	<0.05 **
		D3	30	3	27	LS-BMD (g/cm^2^)	0.91 ± 0.09	0.96 ± 0.10	<0.05 **
		Placebo	30	2	28		0.89 ± 0.07	0.94 ± 0.08	<0.05 **
Mieczkowski, 2014 [23]	12	D3	8	NR	NR	Z score, radius	NSR ^1^	NSR ^1^	ns **
		Standard care	11	NR	NR		NSR ^1^	NSR ^1^	ns **
		D3	8	NR	NR	Z score L1–L4	NSR ^1^	NSR ^1^	ns **
		Standard care	11	NR	NR		NSR ^1^	NSR ^1^	ns **
		D3	8	NR	NR	Z score, femur	NSR ^1^	NSR ^1^	ns **
		Standard care	11	NR	NR		NSR ^1^	NSR ^1^	ns **
Ulrich, 2021 [41]	3	D3	19	4	15	Testosterone (nmol/L)	8.0 ± 3.7 (M) 1.3 ± 1.0 (F)	7.8 ± 3.8 (M) 1.2 ± 1.0 (F)	ns **
		Placebo	19	1	18		11.9 ± 5.0 (M) 0.8 ± 0.5 (F)	11.6 ± 4.0 (M) 0.7 ± 0.4 (F)	ns **

IRR: incidence rate ratio; F: female participants; FN-BMD: femoral neck bone mineral density; LA-BMD: lumbar spine bone mineral density; M: male participants; ref: reference; VAS: visual analog scale; NSR: data not specifically reported. ^1^ Data presented in graphical form only. * *p* value represents the significance level of the reported treatment effect between groups; ** *p* value represents treatment effect within groups from baseline.

**Table 8:** Vitamin D supplementation and ESKD-related outcomes (EPO dosing, AVF maturation, and pruritus).

Author, Year	Length of Follow-Up (mo)	Study Arms	Randomized (n)	Lost to Follow-Up (n)	Included in Final Analysis (n)	Outcome Details	Outcome Measurements		*p* Value
							Baseline	End of Follow-Up	
Ayub, 2022 [30]	2	D3	35	NR	NR	Hb (g/dL)	10.1 ± 1.4	10.4 ± 1.4	0.503 *
		Placebo	35	NR	NR		10.2 ± 1.6	10.2 ± 1.6	
Gregorio, 2021 [32]	6	D3	18	6	12	Hb (g/dL)	11.8 ± 1.1	11.6 ± 0.7	Ns *
		Placebo	14	3	11		12.0 ± 1.3	12.1 ± 1.4	
Mehortotra ^1^, 2013 [20]	6	D3	51		NR	EPO dose (mcg/week)	40	25	0.028 **
		Standard care	28		19		50	NR	ns **
		D3	51		NR	Hb (g/dL)	11.8	11.2	0.17 **
		Standard care	28		19		11.5	NR	ns **
Miskulin, 2016 [28]	6	D2	137	15	122	EPO dose (units/week)	5800 [2600, 12,200]	7000 [2500, 16,000]	0.78 *
		Placebo	139	9	130		5400 [2400, 11,500]	6050 [2000, 11,800]	
Naini, 2015 [36]	4	VD	32	0	22	EPO dose (units/week)	NR ^3^	NR	<0.001 **
		Placebo	32	0	22		NR	NR	ns **
		VD-male	16	0	16	Hb (mg/dL)	9.8 ±1.6	10.6 ±1.1	Ns *
		VD-female	16	0	16		10.1 ± 1.7	11.2 ± 1.2	
		Placebo-male	16	0	16		9.2 ±1.4	10.1 ±0.8	
		Placebo-female	16	0	16		9.2 ± 1.5	10.2 ± 0.9	
Shirazian, 2013 [39]	3	D3	25	0	25	Change in pruritus survey score	-	−38.9%	0.34 *
		Placebo	25	0	25		-	−47.5%	
Wasse, 2014 [24]	0.75 ^2^	D3	25	5	20	% Successful AVF/AVG use at 6 mo	-	45%	0.8 *
		Placebo	27	3	24		-	54%	
		D3	25	5	20	% AVF maturation	-	41%	0.7 *
		Placebo	27	3	24		-	50%	

AVF: arteriovenous fistula; AVG: arteriovenous graft; EPO: erythropoietin; Hb: hemoglobin; VD: vitamin D. ^1^ Abstract only; ^2^ follow-up was 6 months following AVF; ^3^ data presented graphically only. * *p* value represents the significance level of the treatment effect between groups; ** *p* value represents the significance of the treatment effect within groups from baseline.

## Data Availability

Studies and methodologies included in this review are publicly available online.

## Supplementary Materials

The following supporting information can be downloaded at: https://www.mdpi.com/article/10.3390/nu15133072/s1, Table S1: Vitamin D Supplementation and Nutritional Outcomes. Table S2: Vitamin D and Well Being (Quality of Life, Pain, Depression). Table S3: Vitamin D Supplementation and Vitamin D Status (nmol/L).

## Appendix A. Total Number of Records Identified for Each Database and Information Source Up to 31 January 2023

Ovid MEDLINE	2272
Ovid Embase	2984
Ovid EBM Reviews for Cochrane CENTRAL	1409
Web of Science Core Collection	2699
medRxiv	186
ProQuest Dissertations and Theses Global	82
Total number of records	9632
Total number of records after removing duplicates in Covidence	6519

## Appendix B. Search Strategy Embase, MEDLINE, Cochrane CENTRAL, Web of Science, ProQuest Dissertations and Theses, medRxiv

**Embase Classic+Embase <1947 to 2023 January 31>**

1 chronic kidney failure/ or “chronic kidney disease-mineral and bone disorder”/ or renal  osteodystrophy/ (131189)

2 end stage renal disease/ (43428)

3 hemodialysis/ or continuous hemodialysis/ or home dialysis/ (128603)

4 kidney graft/ (50037)

5 glomerulonephritis/ or acute glomerulonephritis/ or allergic glomerulonephritis/ or  chronic glomerulonephritis/ or experimental glomerulonephritis/ or experimental  autoimmune glomerulonephritis/ or focal glomerulonephritis/ or immunoglobulin a  nephropathy/ or immunoglobulin m nephropathy/ or masugi nephritis/ or  membranoproliferative glomerulonephritis/ or membranous glomerulonephritis/ or  minimal change glomerulonephritis/ or proliferative glomerulonephritis/ or rapidly  progressive glomerulonephritis/ (71602)

6 diabetic nephropathy/ or experimental diabetic nephropathy/ or streptozotocin-induced  diabetic nephropathy/ (49751)

7 chronic kidney disease*.mp. [mp=title, abstract, heading word, drug trade name, original  title, device manufacturer, drug manufacturer, device trade name, keyword heading word,  floating subheading word, candidate term word] (119278)

8 CKD.mp. [mp=title, abstract, heading word, drug trade name, original title, device  manufacturer, drug manufacturer, device trade name, keyword heading word, floating  subheading word, candidate term word] (69868)

9 chronic kidney failure.mp. [mp=title, abstract, heading word, drug trade name, original  title, device manufacturer, drug manufacturer, device trade name, keyword heading word,  floating subheading word, candidate term word] (127722)

10 chronic kidney disorder*.mp. [mp=title, abstract, heading word, drug trade name, original  title, device manufacturer, drug manufacturer, device trade name, keyword heading word,  floating subheading word, candidate term word] (69)

11 chronic kidney insufficienc*.mp. [mp=title, abstract, heading word, drug trade name,  original title, device manufacturer, drug manufacturer, device trade name, keyword  heading word, floating subheading word, candidate term word] (253)

12 chronic kidney dysfunction*.mp. [mp=title, abstract, heading word, drug trade name,  original title, device manufacturer, drug manufacturer, device trade name, keyword  heading word, floating subheading word, candidate term word] (134)

13 chronic renal disease*.mp. [mp=title, abstract, heading word, drug trade name, original  title, device manufacturer, drug manufacturer, device trade name, keyword heading word,  floating subheading word, candidate term word] (6945)

14 chronic renal failure.mp. [mp=title, abstract, heading word, drug trade name, original  title, device manufacturer, drug manufacturer, device trade name, keyword heading word,  floating subheading word, candidate term word] (35629)

15 chronic renal insufficienc*.mp. [mp=title, abstract, heading word, drug trade name,  original title, device manufacturer, drug manufacturer, device trade name, keyword  heading word, floating subheading word, candidate term word] (7627)

16 chronic renal disorder*.mp. [mp=title, abstract, heading word, drug trade name, original  title, device manufacturer, drug manufacturer, device trade name, keyword heading word,  floating subheading word, candidate term word] (87)

17 chronic renal dysfunction*.mp. [mp=title, abstract, heading word, drug trade name,  original title, device manufacturer, drug manufacturer, device trade name, keyword  heading word, floating subheading word, candidate term word] (507)

18 nephropath*.mp. [mp=title, abstract, heading word, drug trade name, original title, device  manufacturer, drug manufacturer, device trade name, keyword heading word, floating  subheading word, candidate term word] (123221)

19 nephritis.mp. [mp=title, abstract, heading word, drug trade name, original title, device  manufacturer, drug manufacturer, device trade name, keyword heading word, floating  subheading word, candidate term word] (65692)

20 End stage kidney disease*.mp. [mp=title, abstract, heading word, drug trade name,  original title, device manufacturer, drug manufacturer, device trade name, keyword  heading word, floating subheading word, candidate term word] (8151)

21 endstage kidney disease*.mp. [mp=title, abstract, heading word, drug trade name,  original title, device manufacturer, drug manufacturer, device trade name, keyword  heading word, floating subheading word, candidate term word] (138)

22 End stage kidney failure.mp. [mp=title, abstract, heading word, drug trade name, original  title, device manufacturer, drug manufacturer, device trade name, keyword heading word,  floating subheading word, candidate term word] (426)

23 End stage renal failure.mp. [mp=title, abstract, heading word, drug trade name, original  title, device manufacturer, drug manufacturer, device trade name, keyword heading word,  floating subheading word, candidate term word] (9221)

24 Endstage renal failure.mp. [mp=title, abstract, heading word, drug trade name, original  title, device manufacturer, drug manufacturer, device trade name, keyword heading word,  floating subheading word, candidate term word] (305)

25 End stage renal insufficienc*.mp. [mp=title, abstract, heading word, drug trade name,  original title, device manufacturer, drug manufacturer, device trade name, keyword  heading word, floating subheading word, candidate term word] (104)

26 end stage renal disease*.mp. [mp=title, abstract, heading word, drug trade name, original  title, device manufacturer, drug manufacturer, device trade name, keyword heading word,  floating subheading word, candidate term word] (78278)

27 endstage renal disease*.mp. [mp=title, abstract, heading word, drug trade name, original  title, device manufacturer, drug manufacturer, device trade name, keyword heading word,  floating subheading word, candidate term word] (1230)

28 ERSD.mp. [mp=title, abstract, heading word, drug trade name, original title, device  manufacturer, drug manufacturer, device trade name, keyword heading word, floating  subheading word, candidate term word] (172)

29 ESKD.mp. [mp=title, abstract, heading word, drug trade name, original title, device  manufacturer, drug manufacturer, device trade name, keyword heading word, floating  subheading word, candidate term word] (3596)

30 end stage renal dysfunction*.mp. [mp=title, abstract, heading word, drug trade name,  original title, device manufacturer, drug manufacturer, device trade name, keyword  heading word, floating subheading word, candidate term word] (49)

31 end stage renal disorder*.mp. [mp=title, abstract, heading word, drug trade name, original  title, device manufacturer, drug manufacturer, device trade name, keyword heading word,  floating subheading word, candidate term word] (20)

32 Hemodialysis.mp. [mp=title, abstract, heading word, drug trade name, original title,  device manufacturer, drug manufacturer, device trade name, keyword heading word,  floating subheading word, candidate term word] (172865)

33 Haemodialysis.mp. [mp=title, abstract, heading word, drug trade name, original title,  device manufacturer, drug manufacturer, device trade name, keyword heading word,  floating subheading word, candidate term word] (23963)

34 blood dialysis.mp. [mp=title, abstract, heading word, drug trade name, original title,  device manufacturer, drug manufacturer, device trade name, keyword heading word,  floating subheading word, candidate term word] (90)

35 peritoneal dialysis.mp. [mp=title, abstract, heading word, drug trade name, original title,  device manufacturer, drug manufacturer, device trade name, keyword heading word,  floating subheading word, candidate term word] (53649)

36 peritoneum dialysis.mp. [mp=title, abstract, heading word, drug trade name, original title,  device manufacturer, drug manufacturer, device trade name, keyword heading word,  floating subheading word, candidate term word] (14)

37 kidney transplant*.mp. [mp=title, abstract, heading word, drug trade name, original title,  device manufacturer, drug manufacturer, device trade name, keyword heading word,  floating subheading word, candidate term word] (164392)

38 kidney graft*.mp. [mp=title, abstract, heading word, drug trade name, original title,  device manufacturer, drug manufacturer, device trade name, keyword heading word,  floating subheading word, candidate term word] (67273)

39 renal transplant*.mp. [mp=title, abstract, heading word, drug trade name, original title,  device  manufacturer, drug manufacturer, device trade name, keyword heading word,  floating subheading word, candidate term word] (74984)

40 renal graft*.mp. [mp=title, abstract, heading word, drug trade name, original title, device  manufacturer, drug manufacturer, device trade name, keyword heading word, floating  subheading word, candidate term word] (7283)

41 glomerulonephritis.mp. [mp=title, abstract, heading word, drug trade name, original title,  device manufacturer, drug manufacturer, device trade name, keyword heading word,  floating subheading word, candidate term word] (70854)

42 diabetic kidney disease*.mp. [mp=title, abstract, heading word, drug trade name, original  title, device manufacturer, drug manufacturer, device trade name, keyword heading word,  floating subheading word, candidate term word] (5622)

43 diabetic glomerulopath*.mp. [mp=title, abstract, heading word, drug trade name, original  title, device manufacturer, drug manufacturer, device trade name, keyword heading word,  floating subheading word, candidate term word] (462)

44 diabetic glomerulosclerosis.mp. [mp=title, abstract, heading word, drug trade name,  original title, device manufacturer, drug manufacturer, device trade name, keyword  heading word, floating subheading word, candidate term word] (809)

45 or/1-44  (717694)

46 vitamin d/ or 24,25 dihydroxyvitamin d/ or 25 hydroxyvitamin d/ or “9,10 secocholesta  5,7,10(19) trien 23 yne 1,3,25 triol”/ or “9,10 secocholesta 5,7,10(19) trien 23 yne 3,25  diol”/  or “9,10 secocholesta 5,7,10(19),16 tetraen 23 yne 1,3,25 triol”/ or “9,10  secocholesta 5,7,10(19),22 tetraene 1,3,25,26 tetrol”/ or ascorbic acid plus fluoride plus  retinol plus vitamin d/ or calcium carbonate plus ferrous fumarate plus vitamin d/ or  calcium phosphate dibasic plus ferrous sulfate plus manganese sulfate plus nicotinic acid  plus riboflavin plus thiamine plus vitamin d/ or dihydrotachysterol/ or lunacalcipol/ or  vitamin d derivative/ (111364)

47 ergocalciferol derivative/ or 1,25 dihydroxy 24 epiergocalciferol/ or 1,25  dihydroxyergocalciferol/ or 1,25,26 trihydroxyergocalciferol/ or 1,25,28  trihydroxyergocalciferol/ or 22,23 dihydroergocalciferol/ or 24,25  dihydroxyergocalciferol/ or 25 hydroxyergocalciferol/ or 25,28 dihydroxyergocalciferol/  or doxercalciferol/ or ergocalciferol/ or paricalcitol/ (14105)

48 colecalciferol derivative/ or 1,23,25 trihydroxycolecalciferol/ or 1,24,25  trihydroxycolecalciferol/ or 1beta,25 dihydroxycolecalciferol/ or “20 epi 22 ethoxy  24a,26a,27a trihomo 9,10 secocholesta 5,7,10(19) trien 23 yne 1alpha,3beta,25 triol”/ or  25,26 dihydroxycolecalciferol/ or alendronic acid plus colecalciferol/ or atocalcitol/ or  becocalcidiol/ or betamethasone dipropionate plus calcipotriol/ or calcifediol/ or  calcipotriol/ or calcium carbonate plus colecalciferol plus ibandronic acid/ or calcium  carbonate plus colecalciferol plus risedronic acid/ or calcium carbonate plus  colecalciferol plus zoledronic acid/ or calcium plus colecalciferol/ or colecalciferol/ or  colecalciferol plus strontium ranelate/ or dihydroxycolecalciferol/ or ecalcidene/ or  eldecalcitol/ or elocalcitol/ or hydroxycolecalciferol/ or inecalcitol/ or lexacalcitol/ or  pefcalcitol/ or secalciferol/ or seocalcitol/ or tisocalcitate/ (40983)

49 vitamin d.mp. [mp=title, abstract, heading word, drug trade name, original title, device  manufacturer, drug manufacturer, device trade name, keyword heading word, floating  subheading word, candidate term word] (148373)

50 cholecalciferol.mp. [mp=title, abstract, heading word, drug trade name, original title,  device manufacturer, drug manufacturer, device trade name, keyword heading word,  floating subheading word, candidate term word] (5104)

51 colecalciferol.mp. [mp=title, abstract, heading word, drug trade name, original title,  device manufacturer, drug manufacturer, device trade name, keyword heading word,  floating subheading word, candidate term word] (27882)

52 ergocalciferol.mp. [mp=title, abstract, heading word, drug trade name, original title,  device manufacturer, drug manufacturer, device trade name, keyword heading word,  floating subheading word, candidate term word] (10978)

53 calcifediol.mp. [mp=title, abstract, heading word, drug trade name, original title, device  manufacturer, drug manufacturer, device trade name, keyword heading word, floating  subheading word, candidate term word] (10528)

54 or/46-53 (176509)

55 Randomized controlled trial/ (709180)

56 Controlled clinical study/ (465872)

57 random$.ti,ab. (1794466)

58 randomization/ (93988)

59 intermethod comparison/ (282516)

60 placebo.ti,ab. (344862)

61 (compare or compared or comparison).ti. (594149)

62 ((evaluated or evaluate or evaluating or assessed or assess) and (compare or compared or  comparing or comparison)).ab. (2494576)

63 (open adj label).ti,ab. (96515)

64 ((double or single or doubly or singly) adj (blind or blinded or blindly)).ti,ab. (261329)

65 double blind procedure/ (196987)

66 parallel group$1.ti,ab. (29307)

67 (crossover or cross over).ti,ab. (117328)

68 ((assign$ or match or matched or allocation) adj5 (alternate or group$1 or intervention$1  or patient$1 or subject$1 or participant$1)).ti,ab. (380433)

69 (assigned or allocated).ti,ab. (448259)

70 (controlled adj7 (study or design or trial)).ti,ab. (409679)

71 (volunteer or volunteers).ti,ab.(273285)

72 human experiment/ (573910)

73 trial.ti. (365254)

74 or/55-73 (5822074)

75 (random$ adj sampl$ adj7 (cross section$ or questionnaire$1 or survey$ or  database$1)).ti,ab. not (comparative study/ or controlled study/ or randomi?ed  controlled.ti,ab.  or randomly assigned.ti,ab.) (9056)

76 Cross-sectional study/ not (randomized controlled trial/ or controlled clinical study/ or  controlled study/ or randomi?ed controlled.ti,ab. or control group$1.ti,ab.) (307449)

77 (((case adj control$) and random$) not randomi?ed controlled).ti,ab. (19748)

78 (Systematic review not (trial or study)).ti. (207844)

79 (nonrandom$ not random$).ti,ab. (17863)

80 “Random field$”.ti,ab. (2693)

81 (random cluster adj3 sampl$).ti,ab. (1433)

82 (review.ab. and review.pt.) not trial.ti. (988850)

83 “we searched”.ab. and (review.ti. or review.pt.) (41601)

84 “update review”.ab. (122)

85 (databases adj4 searched).ab. (50313)

86 (rat or rats or mouse or mice or swine or porcine or murine or sheep or lambs or pigs or  piglets  or rabbit or rabbits or cat or cats or dog or dogs or cattle or bovine or monkey or monkeys or  trout or marmoset$1).ti. and animal experiment/ (1147757)

87 Animal experiment/ not (human experiment/ or human/) (2412365)

88 or/75-87 (3952717)

89 74 not 88 (5166159)

90 45 and 54 and 89 (2935)

91 90 not ((exp infant/ or exp child/ or adolescent/) not exp adult/) (2786)

92 limit 91 to dc=20220503-20230131 198

**Ovid MEDLINE(R) ALL <1946 to 31 January 2023>**

1 renal insufficiency, chronic/ or kidney failure, chronic/ or frasier syndrome/ or “chronic  kidney disease-mineral and bone disorder”/ (129960)

2 renal dialysis/ or hemodiafiltration/ or hemodialysis, home/ or peritoneal dialysis/ or  peritoneal dialysis, continuous ambulatory/ (122014)

3 Kidney Transplantation/ (102302)

4 glomerulonephritis/ or anti-glomerular basement membrane disease/ or  glomerulonephritis, iga/ or glomerulonephritis, membranoproliferative/ or  glomerulonephritis, membranous/ or glomerulosclerosis, focal segmental/ or lupus  nephritis/ (50352)

5 Diabetic Nephropathies/ (28066)

6 chronic kidney disease*.mp. [mp=title, abstract, original title, name of substance word,  subject heading word, floating sub-heading word, keyword heading word, organism  supplementary concept word, protocol supplementary concept word, rare disease  supplementary concept word, unique identifier, synonyms] (67154)

7 CKD.mp. [mp=title, abstract, original title, name of substance word, subject heading  word, floating sub-heading word, keyword heading word, organism supplementary  concept word, protocol supplementary concept word, rare disease supplementary concept  word, unique identifier, synonyms] (37708)

8 chronic kidney failure.mp. [mp=title, abstract, original title, name of substance word,  subject heading word, floating sub-heading word, keyword heading word, organism  supplementary concept word, protocol supplementary concept word, rare disease  supplementary concept word, unique identifier, synonyms] (1576)

9 chronic kidney disorder*.mp. [mp=title, abstract, original title, name of substance word,  subject heading word, floating sub-heading word, keyword heading word, organism  supplementary concept word, protocol supplementary concept word, rare disease  supplementary concept word, unique identifier, synonyms] (48)

10 chronic kidney insufficienc*.mp. [mp=title, abstract, original title, name of substance  word, subject heading word, floating sub-heading word, keyword heading word,  organism supplementary concept word, protocol supplementary concept word, rare  disease supplementary concept word, unique identifier, synonyms] (239)

11 chronic kidney dysfunction*.mp. [mp=title, abstract, original title, name of substance  word, subject heading word, floating sub-heading word, keyword heading word,  organism supplementary concept word, protocol supplementary concept word, rare  disease supplementary concept word, unique identifier, synonyms] (80)

12 chronic renal disease*.mp. [mp=title, abstract, original title, name of substance word,  subject heading word, floating sub-heading word, keyword heading word, organism  supplementary concept word, protocol supplementary concept word, rare disease  supplementary concept word, unique identifier, synonyms] (4125)

13 chronic renal failure.mp. [mp=title, abstract, original title, name of substance word,  subject heading word, floating sub-heading word, keyword heading word, organism  supplementary concept word, protocol supplementary concept word, rare disease  supplementary concept word, unique identifier, synonyms] (24429)

14 chronic renal insufficienc*.mp. [mp=title, abstract, original title, name of substance word,  subject heading word, floating sub-heading word, keyword heading word, organism  supplementary concept word, protocol supplementary concept word, rare disease  supplementary concept word, unique identifier, synonyms] (5305)

15 chronic renal disorder*.mp. [mp=title, abstract, original title, name of substance word,  subject heading word, floating sub-heading word, keyword heading word, organism  supplementary concept word, protocol supplementary concept word, rare disease  supplementary concept word, unique identifier, synonyms] (54)

16 chronic renal dysfunction*.mp. [mp=title, abstract, original title, name of substance word,  subject heading word, floating sub-heading word, keyword heading word, organism  supplementary concept word, protocol supplementary concept word, rare disease  supplementary concept word, unique identifier, synonyms] (279)

17 nephropath*.mp. [mp=title, abstract, original title, name of substance word, subject  heading word, floating sub-heading word, keyword heading word, organism  supplementary concept word, protocol supplementary concept word, rare disease  supplementary concept word, unique identifier, synonyms] (77135)

18 nephritis.mp. [mp=title, abstract, original title, name of substance word, subject heading  word, floating sub-heading word, keyword heading word, organism supplementary  concept word, protocol supplementary concept word, rare disease supplementary concept  word, unique identifier, synonyms] (36071)

19 End stage kidney disease*.mp. [mp=title, abstract, original title, name of substance word,  subject heading word, floating sub-heading word, keyword heading word, organism  supplementary concept word, protocol supplementary concept word, rare disease  supplementary concept word, unique identifier, synonyms] (5300)

20 endstage kidney disease*.mp. [mp=title, abstract, original title, name of substance word,  subject heading word, floating sub-heading word, keyword heading word, organism  supplementary concept word, protocol supplementary concept word, rare disease  supplementary concept word, unique identifier, synonyms] (27)

21 End stage kidney failure.mp. [mp=title, abstract, original title, name of substance word,  subject heading word, floating sub-heading word, keyword heading word, organism  supplementary concept word, protocol supplementary concept word, rare disease  supplementary concept word, unique identifier, synonyms] (272)

22 End stage renal failure.mp. [mp=title, abstract, original title, name of substance word,  subject heading word, floating sub-heading word, keyword heading word, organism  supplementary concept word, protocol supplementary concept word, rare disease  supplementary concept word, unique identifier, synonyms] (6410)

23 Endstage renal failure.mp. [mp=title, abstract, original title, name of substance word,  subject heading word, floating sub-heading word, keyword heading word, organism  supplementary concept word, protocol supplementary concept word, rare disease  supplementary concept word, unique identifier, synonyms] (147)

24 End stage renal insufficienc*.mp. [mp=title, abstract, original title, name of substance  word, subject heading word, floating sub-heading word, keyword heading word,  organism supplementary concept word, protocol supplementary concept word, rare  disease supplementary concept word, unique identifier, synonyms] (77)

25 end stage renal disease*.mp. [mp=title, abstract, original title, name of substance word,  subject heading word, floating sub-heading word, keyword heading word, organism  supplementary concept word, protocol supplementary concept word, rare disease  supplementary concept word, unique identifier, synonyms] (37041)

26 endstage renal disease*.mp. [mp=title, abstract, original title, name of substance word,  subject heading word, floating sub-heading word, keyword heading word, organism  supplementary concept word, protocol supplementary concept word, rare disease  supplementary concept word, unique identifier, synonyms] (379)

27 ERSD.mp. [mp=title, abstract, original title, name of substance word, subject heading  word, floating sub-heading word, keyword heading word, organism supplementary  concept word, protocol supplementary concept word, rare disease supplementary concept  word, unique identifier, synonyms] (78)

28 ESKD.mp. [mp=title, abstract, original title, name of substance word, subject heading  word, floating sub-heading word, keyword heading word, organism supplementary  concept word, protocol supplementary concept word, rare disease supplementary concept  word, unique identifier, synonyms] (2037)

29 end stage renal dysfunction*.mp. [mp=title, abstract, original title, name of substance  word, subject heading word, floating sub-heading word, keyword heading word,  organism supplementary concept word, protocol supplementary concept word, rare  disease supplementary concept word, unique identifier, synonyms] (28)

30 end stage renal disorder*.mp. [mp=title, abstract, original title, name of substance word,  subject heading word, floating sub-heading word, keyword heading word, organism  supplementary concept word, protocol supplementary concept word, rare disease  supplementary concept word, unique identifier, synonyms] (11)

31 Hemodialysis.mp. [mp=title, abstract, original title, name of substance word, subject  heading word, floating sub-heading word, keyword heading word, organism  supplementary concept word, protocol supplementary concept word, rare disease  supplementary concept word, unique identifier, synonyms] (71722)

32 Haemodialysis.mp. [mp=title, abstract, original title, name of substance word, subject  heading word, floating sub-heading word, keyword heading word, organism  supplementary concept word, protocol supplementary concept word, rare disease  supplementary concept word, unique identifier, synonyms] (16153)

33 blood dialysis.mp. [mp=title, abstract, original title, name of substance word, subject  heading word, floating sub-heading word, keyword heading word, organism  supplementary concept word, protocol supplementary concept word, rare disease  supplementary concept word, unique identifier, synonyms] (56)

34 peritoneal dialysis.mp. [mp=title, abstract, original title, name of substance word, subject  heading word, floating sub-heading word, keyword heading word, organism  supplementary concept word, protocol supplementary concept word, rare disease  supplementary concept word, unique identifier, synonyms] (34493)

35 kidney transplant*.mp. [mp=title, abstract, original title, name of substance word, subject  heading word, floating sub-heading word, keyword heading word, organism  supplementary concept word, protocol supplementary concept word, rare disease  supplementary concept word, unique identifier, synonyms] (112654)

36 kidney graft*.mp. [mp=title, abstract, original title, name of substance word, subject  heading word, floating sub-heading word, keyword heading word, organism  supplementary concept word, protocol supplementary concept word, rare disease  supplementary concept word, unique identifier, synonyms] (4299)

37 renal transplant*.mp. [mp=title, abstract, original title, name of substance word, subject  heading word, floating sub-heading word, keyword heading word, organism  supplementary concept word, protocol supplementary concept word, rare disease  supplementary concept word, unique identifier, synonyms] (48393)

38 renal graft*.mp. [mp=title, abstract, original title, name of substance word, subject  heading word, floating sub-heading word, keyword heading word, organism  supplementary concept word, protocol supplementary concept word, rare disease  supplementary concept word, unique identifier, synonyms] (3314)

39 glomerulonephritis.mp. [mp=title, abstract, original title, name of substance word, subject  heading word, floating sub-heading word, keyword heading word, organism  supplementary concept word, protocol supplementary concept word, rare disease  supplementary concept word, unique identifier, synonyms] (48450)

40 diabetic kidney disease*.mp. [mp=title, abstract, original title, name of substance word,  subject heading word, floating sub-heading word, keyword heading word, organism  supplementary concept word, protocol supplementary concept word, rare disease  supplementary concept word, unique identifier, synonyms] (3854)

41 diabetic glomerulopath*.mp. [mp=title, abstract, original title, name of substance word,  subject heading word, floating sub-heading word, keyword heading word, organism  supplementary concept word, protocol supplementary concept word, rare disease  supplementary concept word, unique identifier, synonyms] (343)

42 diabetic glomerulosclerosis.mp. [mp=title, abstract, original title, name of substance  word, subject heading word, floating sub-heading word, keyword heading word,  organism supplementary concept word, protocol supplementary concept word, rare  disease supplementary concept word, unique identifier, synonyms] (555)

43 or/1-42 (466945)

44 vitamin d/ or cholecalciferol/ or hydroxycholecalciferols/ or calcifediol/ or  dihydroxycholecalciferols/ or 24,25-dihydroxyvitamin d 3/ or ergocalciferols/ or  dihydrotachysterol/ or 25-hydroxyvitamin d 2/ (54687)

45 vitamin d.mp. [mp=title, abstract, original title, name of substance word, subject heading  word, floating sub-heading word, keyword heading word, organism supplementary  concept word, protocol supplementary concept word, rare disease supplementary concept  word, unique identifier, synonyms] (81191)

46 cholecalciferol.mp. [mp=title, abstract, original title, name of substance word, subject  heading word, floating sub-heading word, keyword heading word, organism  supplementary concept word, protocol supplementary concept word, rare disease  supplementary concept word, unique identifier, synonyms] (9668)

47 colecalciferol.mp. [mp=title, abstract, original title, name of substance word, subject  heading word, floating sub-heading word, keyword heading word, organism  supplementary concept word, protocol supplementary concept word, rare disease  supplementary concept word, unique identifier, synonyms] (82)

48 ergocalciferol.mp. [mp=title, abstract, original title, name of substance word, subject  heading word, floating sub-heading word, keyword heading word, organism  supplementary concept word, protocol supplementary concept word, rare disease  supplementary concept word, unique identifier, synonyms] (803)

49 calcifediol.mp. [mp=title, abstract, original title, name of substance word, subject heading  word, floating sub-heading word, keyword heading word, organism supplementary  concept word, protocol supplementary concept word, rare disease supplementary concept  word, unique identifier, synonyms] (4430)

50 or/44-49 (89029)

51 randomized controlled trial.pt. (566697)

52 controlled clinical trial.pt. (94847)

53 random*.ab. (1273269)

54 placebo.ab. (227712)

55 drug therapy.fs (2482838)

56 trial.ab.(597367)

57 groups.ab. (2342735)

58 or/51-57 (5582292)

59 exp animals/ not humans.sh. (4999748)

60 58 not 59 (4864572)

61 43 and 50 and 60 (3116)

62 61 not ((exp infant/ or exp child/ or adolescent/) not exp adult/) (2884)

63 review.pt. not trial.ti.

64 62 not 63 (2204)

65 limit 64 to dt=20220503-20230131 68

**EBM Reviews—Cochrane Central Register of Controlled Trials <December 2022>**

1 renal insufficiency, chronic/ or kidney failure, chronic/ or frasier syndrome/ or “chronic  kidney disease-mineral and bone disorder”/ (7406)

2 renal dialysis/ or hemodiafiltration/ or hemodialysis, home/ or peritoneal dialysis/ or  peritoneal dialysis, continuous ambulatory/ (5482)

3 Kidney Transplantation/ (3705)

4 glomerulonephritis/ or anti-glomerular basement membrane disease/ or  glomerulonephritis, iga/ or glomerulonephritis, membranoproliferative/ or  glomerulonephritis, membranous/ or glomerulosclerosis, focal segmental/ or lupus  nephritis/ (963)

5 Diabetic Nephropathies/ (1526)

6 chronic kidney disease*.mp. [mp=title, original title, abstract, floating sub-heading word,  mesh headings, heading words, keyword] (9193)

7 CKD.mp. [mp=title, original title, abstract, floating sub-heading word, mesh headings,  heading words, keyword] (6724)

8 chronic kidney failure.mp. [mp=title, original title, abstract, floating sub-heading word,  mesh headings, heading words, keyword] (4061)

9 chronic kidney disorder*.mp. [mp=title, original title, abstract, floating sub-heading  word, mesh headings, heading words, keyword] (4)

10 chronic kidney insufficienc*.mp. [mp=title, original title, abstract, floating sub-heading  word, mesh headings, heading words, keyword] (9)

11 chronic kidney dysfunction*.mp. [mp=title, original title, abstract, floating sub-heading  word, mesh headings, heading words, keyword] (15)

12 chronic renal disease*.mp. [mp=title, original title, abstract, floating sub-heading word,  mesh headings, heading words, keyword] (376)

13 chronic renal failure.mp. [mp=title, original title, abstract, floating sub-heading word,  mesh headings, heading words, keyword] (2159)

14 chronic renal insufficienc*.mp. [mp=title, original title, abstract, floating sub-heading  word, mesh headings, heading words, keyword] (453)

15 chronic renal disorder*.mp. [mp=title, original title, abstract, floating sub-heading word,  mesh headings, heading words, keyword] (1)

16 chronic renal dysfunction*.mp. [mp=title, original title, abstract, floating sub-heading  word, mesh headings, heading words, keyword] (44)

17 nephropath*.mp. [mp=title, original title, abstract, floating sub-heading word, mesh  headings, heading words, keyword] (8376)

18 nephritis.mp. [mp=title, original title, abstract, floating sub-heading word, mesh  headings, heading words, keyword] (1535)

19 End stage kidney disease*.mp. [mp=title, original title, abstract, floating sub-heading  word, mesh headings, heading words, keyword] (655)

20 endstage kidney disease*.mp. [mp=title, original title, abstract, floating sub-heading  word, mesh headings, heading words, keyword] (11)

21 End stage kidney failure.mp. [mp=title, original title, abstract, floating sub-heading word,  mesh headings, heading words, keyword] (30)

22 End stage renal failure.mp. [mp=title, original title, abstract, floating sub-heading word,  mesh headings, heading words, keyword] (526)

23 Endstage renal failure.mp. [mp=title, original title, abstract, floating sub-heading word,  mesh headings, heading words, keyword] (11)

24 End stage renal insufficienc*.mp. [mp=title, original title, abstract, floating sub-heading  word, mesh headings, heading words, keyword] (4)

25 end stage renal disease*.mp. [mp=title, original title, abstract, floating sub-heading word,  mesh headings, heading words, keyword] (4710)

26 endstage renal disease*.mp. [mp=title, original title, abstract, floating sub-heading word,  mesh headings, heading words, keyword] (55)

27 ERSD.mp. [mp=title, original title, abstract, floating sub-heading word, mesh headings,  heading words, keyword] (9)

28 ESKD.mp. [mp=title, original title, abstract, floating sub-heading word, mesh headings,  heading words, keyword] (248)

29 end stage renal dysfunction*.mp. [mp=title, original title, abstract, floating sub-heading  word, mesh headings, heading words, keyword] (3)

30 end stage renal disorder*.mp. [mp=title, original title, abstract, floating sub-heading  word, mesh headings, heading words, keyword] (0)

31 Hemodialysis.mp. [mp=title, original title, abstract, floating sub-heading word, mesh  headings, heading words, keyword] (12089)

32 Haemodialysis.mp. [mp=title, original title, abstract, floating sub-heading word, mesh  headings, heading words, keyword] (2293)

33 blood dialysis.mp. [mp=title, original title, abstract, floating sub-heading word, mesh  headings, heading words, keyword] (13)

34 peritoneal dialysis.mp. [mp=title, original title, abstract, floating sub-heading word, mesh  headings, heading words, keyword] (2493)

35 kidney transplant*.mp. [mp=title, original title, abstract, floating sub-heading word, mesh  headings, heading words, keyword] (8662)

36 kidney graft*.mp. [mp=title, original title, abstract, floating sub-heading word, mesh  headings, heading words, keyword] (2638)

37 renal transplant*.mp. [mp=title, original title, abstract, floating sub-heading word, mesh  headings, heading words, keyword] (5881)

38 renal graft*.mp. [mp=title, original title, abstract, floating sub-heading word, mesh  headings, heading words, keyword] (305)

39 glomerulonephritis.mp. [mp=title, original title, abstract, floating sub-heading word,  mesh headings, heading words, keyword] (1512)

40 diabetic kidney disease*.mp. [mp=title, original title, abstract, floating sub-heading word,  mesh headings, heading words, keyword] (508)

41 diabetic glomerulopath*.mp. [mp=title, original title, abstract, floating sub-heading word,  mesh headings, heading words, keyword] (21)

42 diabetic glomerulosclerosis.mp. [mp=title, original title, abstract, floating sub-heading  word, mesh headings, heading words, keyword] (7)

43 or/1-42 (46124)

44 vitamin d/ or cholecalciferol/ or hydroxycholecalciferols/ or calcifediol/ or  dihydroxycholecalciferols/ or 24,25-dihydroxyvitamin d 3/ or ergocalciferols/ or  dihydrotachysterol/ or 25-hydroxyvitamin d 2/ (5300)

45 vitamin d.mp. [mp=title, original title, abstract, floating sub-heading word, mesh  headings, heading words, keyword] (13641)

46 cholecalciferol.mp. [mp=title, original title, abstract, floating sub-heading word, mesh  headings, heading words, keyword] (3264)

47 colecalciferol.mp. [mp=title, original title, abstract, floating sub-heading word, mesh  headings, heading words, keyword] (978)

48 ergocalciferol.mp. [mp=title, original title, abstract, floating sub-heading word, mesh  headings, heading words, keyword] (328)

49 calcifediol.mp. [mp=title, original title, abstract, floating sub-heading word, mesh  headings, heading words, keyword] (610)

50 or/44-49 (14411)

51 43 and 50 (1347)

52 51 not ((exp infant/ or exp child/ or adolescent/) not exp adult/) (1321)

53 limit 52 to up=202205-202301 88

**Web of Science**

**#1**  TS=(volunteer or volunteers)

**#2**  TS=((controlled) NEAR/7 (study or design or trial))

**#3**  TS=(assigned or allocated)

**#4**  TS=((assign or assigns or match or matched or allocation) NEAR/5 (alternate or group or  groups or intervention or interventions or patient or patients or subject or subjects or  participant or participants))

**#5**  TS=((double or single or doubly or singly) NEAR (blind or blinded or blindly))

**#6**   TS=(open NEAR label)

**#7**  AB=((evaluated or evaluate or evaluating or assessed or assess) and (compare or  compared or comparing or comparison))

**#8**  TI=(compare or compared or comparison)

**#9**  AB=groups

**#10**  ((TS=(“controlled clinical” OR “control group” OR trial or placebo OR “drug therap*”  OR random* OR “parallel group” OR “parallel groups” OR crossover OR “cross over”)))

**#11**  #10 OR #9 OR #8 OR #7 OR #6 OR #5 OR #4 OR #3 OR #2 OR #1

**#12**  ((TS=(“chronic kidney disease*” OR CKD OR “chronic kidney failure” OR “chronic  kidney disorder*” OR “chronic kidney insufficienc*” OR “chronic kidney dysfunction*”  OR “chronic renal disease*” OR “chronic renal failure” OR “chronic renal insufficienc*”  OR “chronic renal disorder*” OR “chronic renal dysfunction*” OR nephropath* OR  nephritis OR “End stage kidney disease*” OR “endstage kidney disease*” OR “End  stage kidney failure” OR “End stage renal failure” OR “Endstage renal failure” OR “End  stage renal insufficienc*” OR “end stage renal disease*” OR “endstage renal disease*”  OR ERSD OR EKSD OR “end stage renal dysfunction*” OR “end stage renal disorder*”  OR Hemodialysis OR Haemodialysis OR “blood dialysis” OR “peritoneal dialysis”  OR “kidney transplant*” OR “kidney graft*” OR “renal transplant*” OR “renal graft*”  OR glomerulonephritis OR “diabetic kidney disease*” OR “diabetic glomerulopath*”  OR “diabetic glomerulosclerosis”)))

**#13**  TS=(“vitamin d” OR cholecalciferol OR colecalciferol OR ergocalciferol OR calcifediol  OR hydroxycholecalciferol* OR dihydroxycholecalciferol* OR dihydrotachysterol)

**#14**  #13 AND #12 AND #11

**#15**  #13 AND #12 AND #11 and Review Articles (Exclude—Document Types) (2581)

**#14**  2022-05-03 to 2023-01-31 (Index Date) (118)

**ProQuest Dissertations & Theses Global**

((ab(“vitamin d”) OR ti(“vitamin d”)) AND (ab((“chronic kidney disease*” OR “chronic renal disease*” OR “end stage kidney disease” OR hemodialysis OR “peritoneal dialysis” OR “kidney transplant”)) OR ti((“chronic kidney disease*” OR “chronic renal disease*” OR “end stage kidney disease” OR hemodialysis OR “peritoneal dialysis” OR “kidney transplant”)))) AND (ab((“chronic kidney disease*” OR “chronic renal disease*” OR “end stage kidney disease” OR hemodialysis OR “peritoneal dialysis” OR “kidney transplant”)) OR ti((“chronic kidney disease*” OR “chronic renal disease*” OR “end stage kidney disease” OR hemodialysis OR “peritoneal dialysis” OR “kidney transplant”)))

82 total results

Searched with filter for 3 May 2022–31 January 2023  0 results

**medRxiv**

Vitamin d

cholecalciferol

colecalciferol

ergocalciferol

calcifediol

hydroxycholecalciferol

dihydroxycholecalciferol

dihydrotachysterol

151 total results

Searched with filter for 3 May 2022–31 January 2023  35 results
