# Supplementary material for: Does Native Vitamin D Supplementation Have Pleiotropic Effects in Patients with End-Stage Kidney Disease? A Systematic Review of Randomized Trials
Source: Nutrients. 2023 Jul 7;15(13):3072. doi: 10.3390/nu15133072 (PMC10346817; doi:10.3390/nu15133072)
Supplement: Supplementary file 1 [file nutrients-15-03072-s001.zip › nutrients-2446082-supplementary.pdf]

Supplemental Material:

Supplemental Table S1. Vitamin D Supplementation and Nutritional Outcomes.

Author, year	Length of Follow-Up (mo)	Study Arms	Randomized (n)	Lost to Follow Up (n)	Included in Final Analysis (n)	Outcome Details	Outcomes Measurements		P value *
							Baseline	End of Follow-Up	
Khajehdehi, 2000 [26]	3	D3	21	6	15	Triglyceride to HDL-c Ratio (mean, SD)	7.35 ±1.26	6.37 ±1.14	0.0001**
		Placebo	21	7	14		7.12 ±1.46	7.71 ±1.34	ns**
		D3	21	6	15	LDL-c/HDL-c	6.59 ±4.55	5.09 ±1.55	ns**
		Placebo	21	7	14		4.66 ±1.63	4.74 ±1.69	ns**
		D3	21	6	15	Cholesterol/ HDL-c ratio	7.65 ±1.83	7.11 ±1.74	ns**
		Placebo	21	7	14		6.94 ±1.75	6.6 ±1.76	ns**
		D3	21	6	15	Triglycerides mmol/L	7.16 ±1.24	6.41 ±1.09	0.001**
		Placebo	21	7	14		6.77 ±1.00	6.65 ±0.88	ns**
		D3	21	6	15	Cholesterol mmol/L	7.42 ±1.45	7.09 ±1.50	ns**
		Placebo	21	7	14		6.54 ±1.09	6.50 ±1.19	ns**
		D3	21	6	15	LDL-c mmol/L	6.57 ±1.11	5.07 ±1.33	ns**
		Placebo	21	7	14		4.37 ±1.17	4.59 ±1.15	ns**
		D3	21	6	15	HDL-c mmol/L	0.98 ±0.14	1.01 ±0.16	**
		Placebo	21	7	14		0.97 ±0.17	1.01 ±0.18	ns**
Ayub, 2022 [30]	2	D3	35			Albumin (g/L)	36.5 ±3.8	36.6 ±4.2	0.818
		Placebo	35				35.7 ±4.4	35.9 ±4.4	
Brimble, 2022 [29]	12	D3	34	5	29	Albumin (g/L)	34.8 ±6.4	34.1 ±5.6	0.2
		Placebo	31	5	24		36.6 ±6.4	32.6 ±9.8	
Gregorio 2021 [32]	6	D3	18	6	12	Albumin (g/L)	39 ±2.0	4.0 ±0.2	ns
		Placebo	14	3	11		41 ±2.0	4.2 ±0.3	
Seirafian, 2014 [38]	3	VD	49	3	46	Albumin (g/L)	34 ±5.2	33 ±4.5	0.38**
		Placebo	40	2	38		33 ±5.3	32 ±6.1	0.21**
Wang, 2016 [15]	12	D3	362	0	362	Albumin (g/L)	35.2 ±5.9	37.3 ±8.7	0.08
		Placebo	364	0	364		34.7 ±4.3	34.52 ±6.	
		D3	362	0	362	Prealbumin (g/L)	323.6 ±42.9	337.9 ±60.1	0.07
		Placebo	364	0	364		341.7 ±55.3	326.5 ±48.5	

HDL-c: high density lipoprotein cholesterol; LDL-c: low density lipoprotein cholesterol. * p value represents the significance level of the treatment effect between groups, ** p value represents the significance level of the treatment effect within groups from baseline

Supplemental Table S2. Vitamin D and Well Being (Quality of Life, Pain, Depression).

Author, Year	Length of Follow-Up (mo)	Study Arms	Randomized (n)	Lost to Follow-Up (n)	Included in Final Analysis (n)	Outcome Details	Outcome Measurements		P value*
							Baseline	End of Follow-Up	
Ayub, 2022 [30]	2	D3	35	NR	NR	Chronic Pain (VAS)	7.09 ±1.42	3.20 ±1.87	<0.001 **
		Placebo	35	NR	NR		6.37 ±1.6	5.37 ±0.004	0.004**
Wang, 2016 [15]	12	D3	373	11	362	BDI-II	22.7 ±4.3	19.6 ±3.7	0.06
		Placebo	373	9	364		21.9 ±5.4	20.8 ±5.1	
Hewit, 2013 [33]	6	D3	30	0	30	KDQOL-36	NR	NR	ns
		Placebo	30	0	30		NR	NR	
Singer, 2019 [40]	12	D3	36	7	29	KDQOL-SF	70.4 ± 13	73 ±15	0.66
		Placebo	32	6	26		73.1 ± 13.8	75 ±14	

BDI-II: Beck's Depressive Inventory-II Chinese version; KDQOL-36: Kidney Disease Quality of Life-36; KDQOL-SF: Kidney Disease Quality of Life Short Form. * p value represents the significance level of the effect of treatment between groups, ** p value represents the significance level of the treatment effect within groups from baseline

Supplemental Table S3. Vitamin D Supplementation and Vitamin D Status (nmol/L).

Author	Baseline 25D		Study End 25D		Hypercalcemia in VD group	Hypercalcemia in control group	Vitamin D toxicity 25(OH)D > 250 nmol/L
	25(OH)D (nmol/L)	Control	Vitamin D	Control			
Ambrus, 2003 [21]	87.4 ±39.9	Not specifically reported[1]	132.3 ± 5	Not specifically reported[1]	12	10	NR
Ayub, 2022 [30]	37.7 ±13.3	42.5 ± 14.0	64.8 ± 17.7	35.1 ± 11.7	NR	NR	NR
Bahn, 2015 [27]	54.7 ± 17.2		25D > 80 nmol/L 91% in weekly arm 65% in monthly arm	25D > 80 nmol/L 35%	Monthly arm: 1 Weekly arm: 2	1	NR
Brimble, 2022 [29]	51.6 ± 29.2	48.5 ± 21.5	68.9 ± 14.3	40.3 ± 17.6	4 (11.8%)	6 (19.4%)	NR
Delayne, 2013 [31]	30± 12	30 ± 15	Not specifically reported[1]	Not specifically reported[1]	0	0	0
Gregoiro, 2021 [32]	37.9 [26.5-57.7]	54.9 [42.9-60.2]	115.1 [97.6-156.8]	62.7 [34.2-77.4]	1	0	NR
Hewitt, 2013 [33]	44.9 ± 12.5	39.9 ± 9.0	89.9 ± 20	39.9 ± 17.5	3	2	NR

Khajehdehi, 2000 [26]	NR	NR	NR	NR	NR	NR	NR
Mehrotra, 2013 [20]	32.2	32.5	97.8	NR	0	NR	NR
Meireles [34]	35.7 ± 11.7	34.7 ± 10.5	107.6 ± 27.5	33.7 ± 10.7	0	0	NR
Mieczkowski, 2014 [23]	28.2 (16.6-48.2)	37.2 (19.7-44.4)	112.1 (77.4-147.3)	44.9 (18.1-65.9)	3	0	NR
Miskulin, 2016 [28]	39.9 ± 14.7	42.2 ± 16.0	97.8 ± 12.2	43.7 ± 18.5	0	0	NR
Morrone, 2021 [22]	29.5 ± 17.0	32.2 ± 16.5	36% achieved 25D > 75 nmol/L	11% achieved 25D > 75 nmol/L	2	8	1 (VD)<br>2 (SC)
Mose, 2014 [35]	28 [20,48]	28 [20,69]	84 [65,125]	30 [22,0]	1	5	NR
Naini, 2015 [36]	M: 54.2±14.9<br>F: 50.0 ± 21.31	M: 52.2 ± 16.2<br>F: 53.9 ±16.2	M: 79.2 ± 7.6<br>F: 79 ± 9.4	M: 54.2 ± 14.9<br>F: 57.7 ± 16.2	NR	NR	NR
Siebert, 2013 [37]	29.4 ± 11.2	33.6 ± 16.6	87.8 ± 22.3	24.6 ± 8.0	0	1	NR
Seirafian, 2014 [38]	42.2 ± 35.9	48.7 ± 63.6	79.6 ± 36.7	30.4 ± 14.0	NR	NR	NR
Shirazian, 2013 [39]	49.2 ± 25.2	38.2 ± 17.5	96.6 ± 33.5	41.7 ± 25.7	0	0	NR
Singer, 2019 [40]	33.9 ± 9.0	33.8 ± 11.2	119 [101-157]	37 [28-57]	9	6	0
Ulrich, 2021 [41]	32.3 ± 17.8		87.8 ± 22.3	29.4 ± 11.2	NR	NR	NR
Wang, 2016 [15]	22.9 ± 4.1	24.2 ± 9.8	41.3 ± 13.7	23.1 ± 7.5	NR	NR	NR
Wasse, 2014 [24]	36.4 ± 14.5	41.9 ± 16.2	133.3 ± 44.2	45.9 ± 18.5	NR	NR	NR
Zheng, 2018 [25]	47.9 ± 18.5	45.4 ± 21.0	93.4 ± 24.0	58.4 ± 18.7	NR	NR	NR

F: female participants; M: male participants; NR: Not reported; VD: Vitamin D; [1] data presented graphically.
